# Caloric restriction creates a metabolic pattern of chronological aging delay that in budding yeast differs from the metabolic design established by two other geroprotectors

**DOI:** 10.18632/oncotarget.27926

**Published:** 2021-03-30

**Authors:** Karamat Mohammad, Vladimir I. Titorenko

**Affiliations:** ^1^Department of Biology, Concordia University, Montreal, Quebec H4B 1R6, Canada

**Keywords:** cellular aging, geroprotectors, caloric restriction, metabolism, methionine

## Abstract

Caloric restriction and the *tor1Δ* mutation are robust geroprotectors in yeast and other eukaryotes. Lithocholic acid is a potent geroprotector in *Saccharomyces* *cerevisiae*. Here, we used liquid chromatography coupled with tandem mass spectrometry method of non-targeted metabolomics to compare the effects of these three geroprotectors on the intracellular metabolome of chronologically aging budding yeast. Yeast cells were cultured in a nutrient-rich medium. Our metabolomic analysis identified and quantitated 193 structurally and functionally diverse water-soluble metabolites implicated in the major pathways of cellular metabolism. We show that the three different geroprotectors create distinct metabolic profiles throughout the entire chronological lifespan of *S. cerevisiae*. We demonstrate that caloric restriction generates a unique metabolic pattern. Unlike the *tor1Δ* mutation or lithocholic acid, it slows down the metabolic pathway for sulfur amino acid biosynthesis from aspartate, sulfate and 5-methyltetrahydrofolate. Consequently, caloric restriction significantly lowers the intracellular concentrations of methionine, *S*-adenosylmethionine and cysteine. We also noticed that the low-calorie diet, but not the *tor1Δ* mutation or lithocholic acid, decreases intracellular ATP, increases the ADP:ATP and AMP:ATP ratios, and rises intracellular ADP during chronological aging. We propose a model of how the specific remodeling of cellular metabolism by caloric restriction contributes to yeast chronological aging delay.

## INTRODUCTION

A body of evidence indicates that metabolism is an essential contributor to the aging and longevity of eukaryotic organisms across phyla. Indeed, healthy aging of the evolutionarily distant eukaryotes coincides with age-related changes in the concentrations of specific metabolites within cells, tissues, organs and biological fluids [1–15]. These changes are considered metabolic biomarkers characteristic of an aging-associated deterioration in cellular functionality, tissue and organs homeostasis, and organismal health [1–15]. Furthermore, such dietary interventions as caloric restriction (CR), reduced protein intake, a limited supply of single amino acid and, alternating cycles of feeding and fasting are robust geroprotectors that specifically rewire cellular and organismal metabolism in various eukaryotic organisms [16–34]. Moreover, allelic variants of the genes implicated in diverse metabolic pathways delay aging and extend longevity in eukaryotic organisms across species [3, 5, 6, 8, 9, 11, 14, 35–43]. Besides, pharmacological interventions that target distinct aspects of metabolism are potent geroprotectors in diverse eukaryotes; these interventions include metformin, rapamycin, resveratrol, spermidine and others [36, 44–58]. Additionally, it has been emphasized that each of the nine common denominators (hallmarks) of aging is linked to a specific remodeling of metabolism [59, 60]. These aging hallmarks include the damage and repair of nuclear DNA, shortening of telomeres, epigenetic regulation changes, proteotoxic stress, deregulation of nutrient sensing, deterioration of mitochondrial functionality, cellular senescence, decline in stem cell number and functionality, and changes in intercellular communications [59]. Based on all these observations, the existence of a metabolic “clock,” “signature,” “footprint” or “profile” of aging delay has been proposed [2, 4–6, 8, 20, 59].

It remained unclear if different dietary, genetic and pharmacological anti-aging interventions set up a similar metabolic pattern of aging delay or each of them generates a distinct metabolic profile. In this study, we used non-targeted metabolomics of chronologically aging budding yeast to clarify this issue. We show that three different geroprotectors create distinct metabolic profiles. We identified a unique metabolic pattern established by one of these geroprotectors.

## RESULTS

### CR, the *tor1Δ* mutation and LCA extend the longevity of chronologically aging yeast

We investigated how efficiently CR, the *tor1Δ* mutation and LCA prolong the longevity of chronologically aging wild-type (WT) strain BY4742. WT cells were cultured in the nutrient-rich YP (1% yeast extract and 2% peptone) medium supplemented with glucose as a sole carbon source. Our previous studies showed that a yeast culture in this nutrient-rich medium provides a beneficial model system for elucidating the chronological aging of multicellular eukaryotes [61].

WT strain culture in the YP medium that initially contained 2% (w/v) glucose served as a control non-CR culture for examining the CR-dependent longevity extension in chronologically aging yeast [61]. A WT strain culture in the same YP medium, but initially containing 0.2% (w/v) glucose, was used as a model system for studying longevity extension by a CR diet [61].

The pro-longevity effect of the *tor1Δ* mutation in the BY4742 genetic background was assessed in the mutant yeast cells cultured in the YP medium supplemented with 2% (w/v) glucose. Under these conditions of culturing, the *tor1Δ* mutation exhibited the highest longevity-extending efficiency [62].

LCA’s greatest beneficial effect on the longevity of chronologically aging WT strain was observed if LCA was used at a final concentration of 50 μM and yeast cells were cultured under CR on 0.2% (w/v) glucose [62]. Under CR conditions on 0.2% (w/v) glucose, a WT strain culture without LCA served as a control for elucidating the LCA-dependent longevity extension.

**Figure 1:**
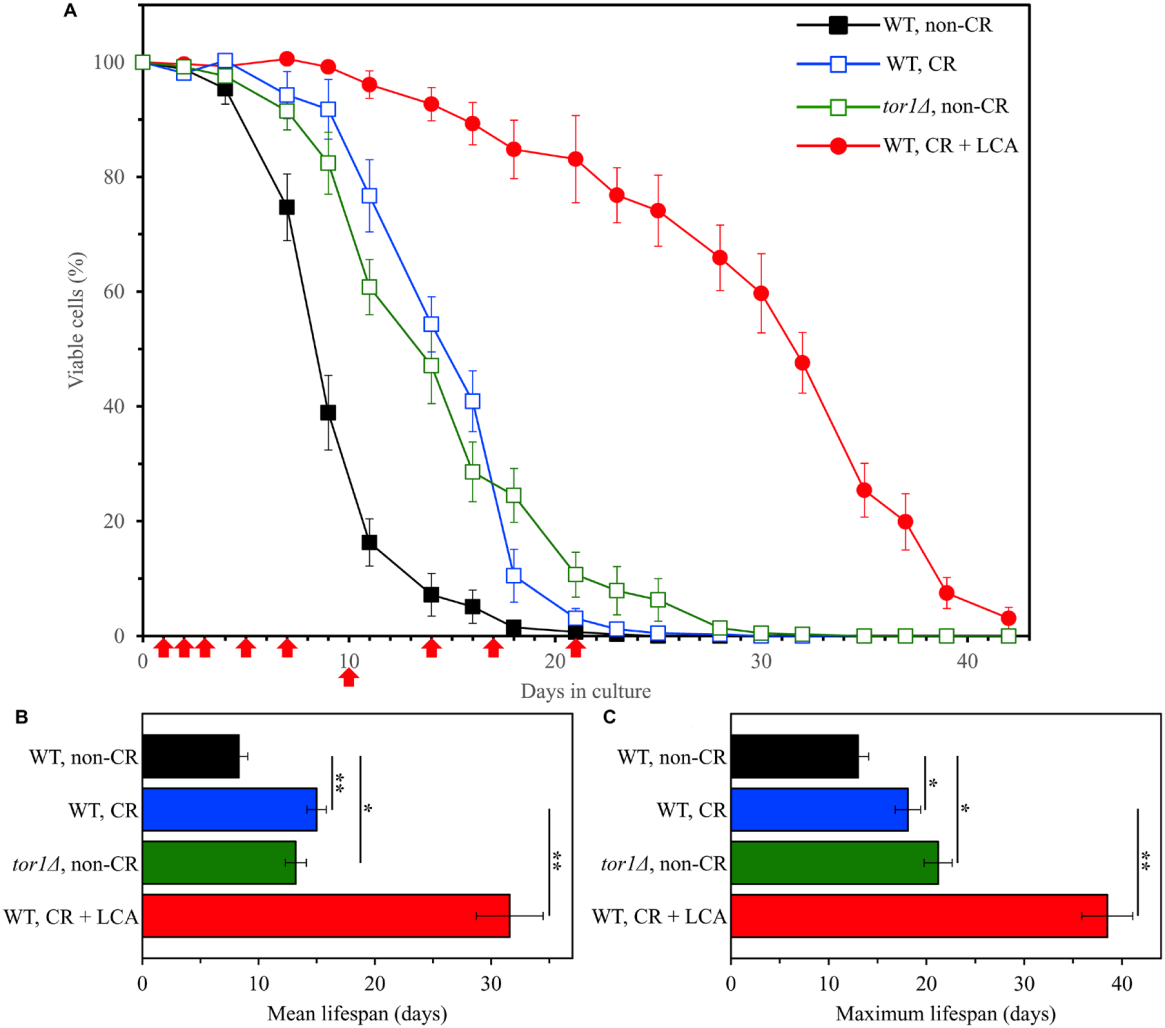
Caloric restriction (CR), the *tor1Δ* mutation and lithocholic acid (LCA) increase the chronological lifespan (CLS) of budding yeast. The wild-type (WT) strain BY4742 was cultured in the nutrient-rich YP medium initially containing 2% (w/v) glucose (non-CR conditions), 0.2% (w/v) glucose (CR conditions) or 0.2% (w/v) glucose and 50 μM LCA (CR + LCA conditions). The *tor1*Δ mutant strain in the BY4742 genetic background was cultured in nutrient-rich YP medium initially containing 2% (w/v) glucose (non-CR conditions). Survival curves (**A**) and the mean and maximum lifespans (**B** and **C**, respectively) of chronologically aging WT and *tor1Δ* cells are shown. Data are presented as means ± SEM (*n* = 3). In graph A, CLS extension was significant for all three longevity-extending interventions tested (*p* < 0.05; the *p value*s for comparing each pair of survival curves were calculated using the logrank test described in Materials and Methods). In graphs B and C, ^*^ *p* < 0.05 and ^**^ *p* < 0.01 (the *p value*s for comparing the means of two groups were calculated using an unpaired two-tailed *t* test described in Materials and Methods). Arrows mark the time points at which cell aliquots were taken for the metabolomic analysis by LC-MS/MS.

We found that CR (a dietary geroprotective intervention), the *tor1Δ* mutation (a genetic geroprotective intervention) and LCA (a pharmacological geroprotective intervention) significantly increase the mean and maximum chronological lifespans (CLS) of WT yeast cultured under the above conditions (Figure 1).

### CR, the *tor1Δ* mutation and LCA create different metabolic patterns throughout the entire chronological lifespan

We used a recently developed liquid chromatography coupled with tandem mass spectrometry (LC-MS/MS) method of non-targeted metabolomics [63] to identify and quantitate the intracellular water-soluble metabolites extracted from chronologically aging yeast. Cell aliquots for the metabolomic analysis by LC-MS/MS were collected on days 1, 2, 3, 5, 7, 10, 14, 17 and 21 of culturing (Figure 1A). A total of 193 metabolites were identified and quantitated in each of the four cultures assessed. These metabolites included AMP, ADP, ATP, FAD^+^, FMN, FADH_2_, NAD^+^, NADH, NADP^+^, NADPH, other nucleotides, amino acids, monosaccharides, intermediates of glycolysis and tricarboxylic cycle intermediates [63].

**Figure 2:**
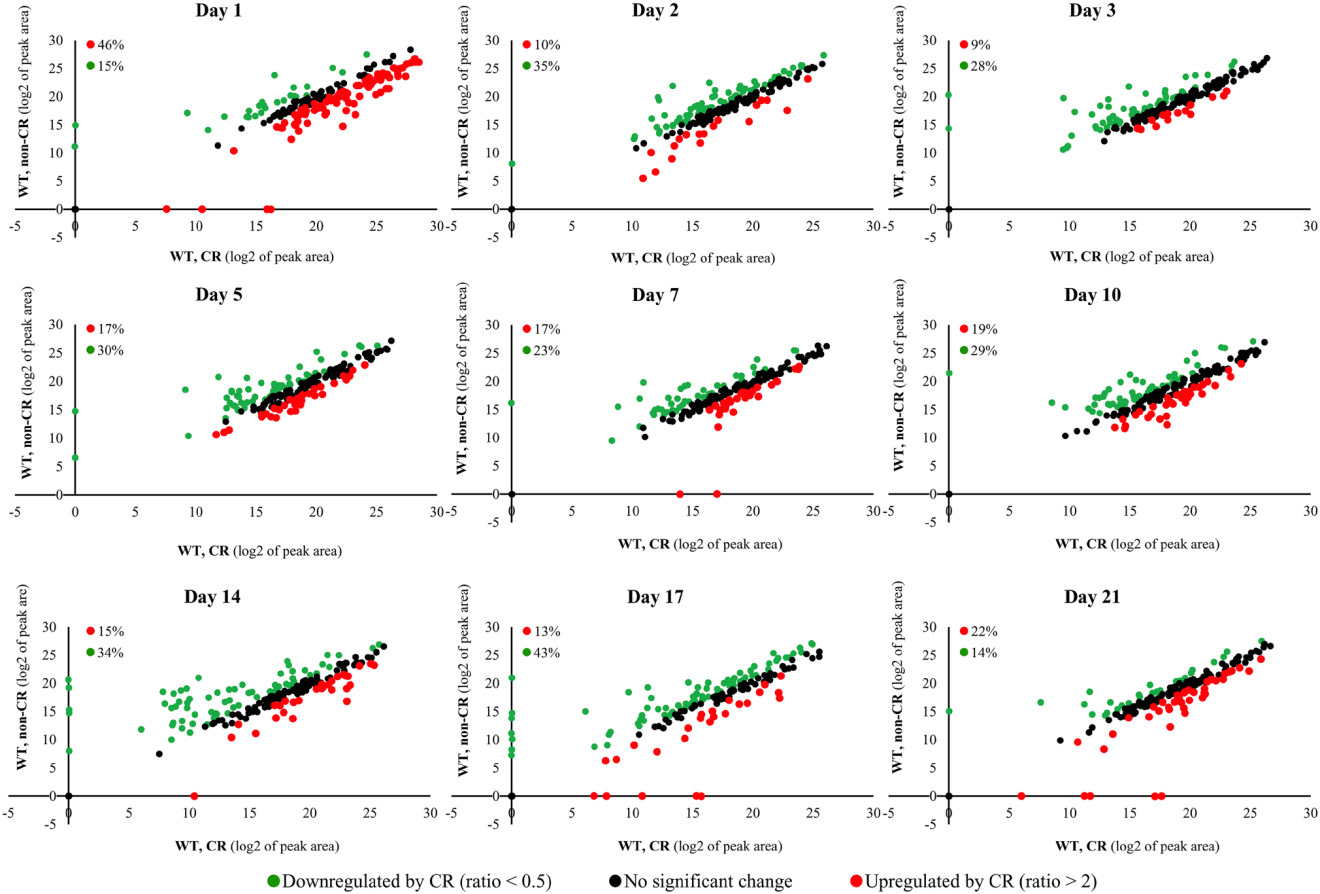
CR causes extensive remodeling of the water-soluble metabolome within WT cells. The WT strain BY4742 was cultured in the nutrient-rich YP medium initially containing 0.2% (w/v) glucose (CR conditions) or 2% (w/v) glucose (non-CR conditions). Cell aliquots for metabolic activity quenching and metabolite extraction were collected on days 1, 2, 3, 5, 7, 10, 14, 17 and 21 of culturing. The use of LC-MS/MS to identify and quantitate the intracellular water-soluble metabolites is described in Materials and Methods. Scatter plots comparing the relative abundance of water-soluble metabolites within WT cells cultured under CR or non-CR conditions are shown. The plots are on a log_2_-log_2_ scale of mass spectrometric peak areas for different metabolites. The percentage abundance of metabolites that were upregulated (ratio > 2; displayed in red) or downregulated (ratio < 0.5; displayed in green) by CR is provided for each time point.

We found that CR causes extensive remodeling of the water-soluble metabolome within WT cells (Figure 2). The CR-dependent remodeling of the water-soluble metabolome was observed throughout the entire chronological lifespan (Figure 2). From 14% to 43% of the identified metabolites were downregulated in WT cells recovered on different days of culturing under CR conditions (Figure 2). Culturing under CR conditions caused the upregulation of many (from 9% to 46%) of metabolites at various stages of the aging process (Figure 2).

**Figure 3:**
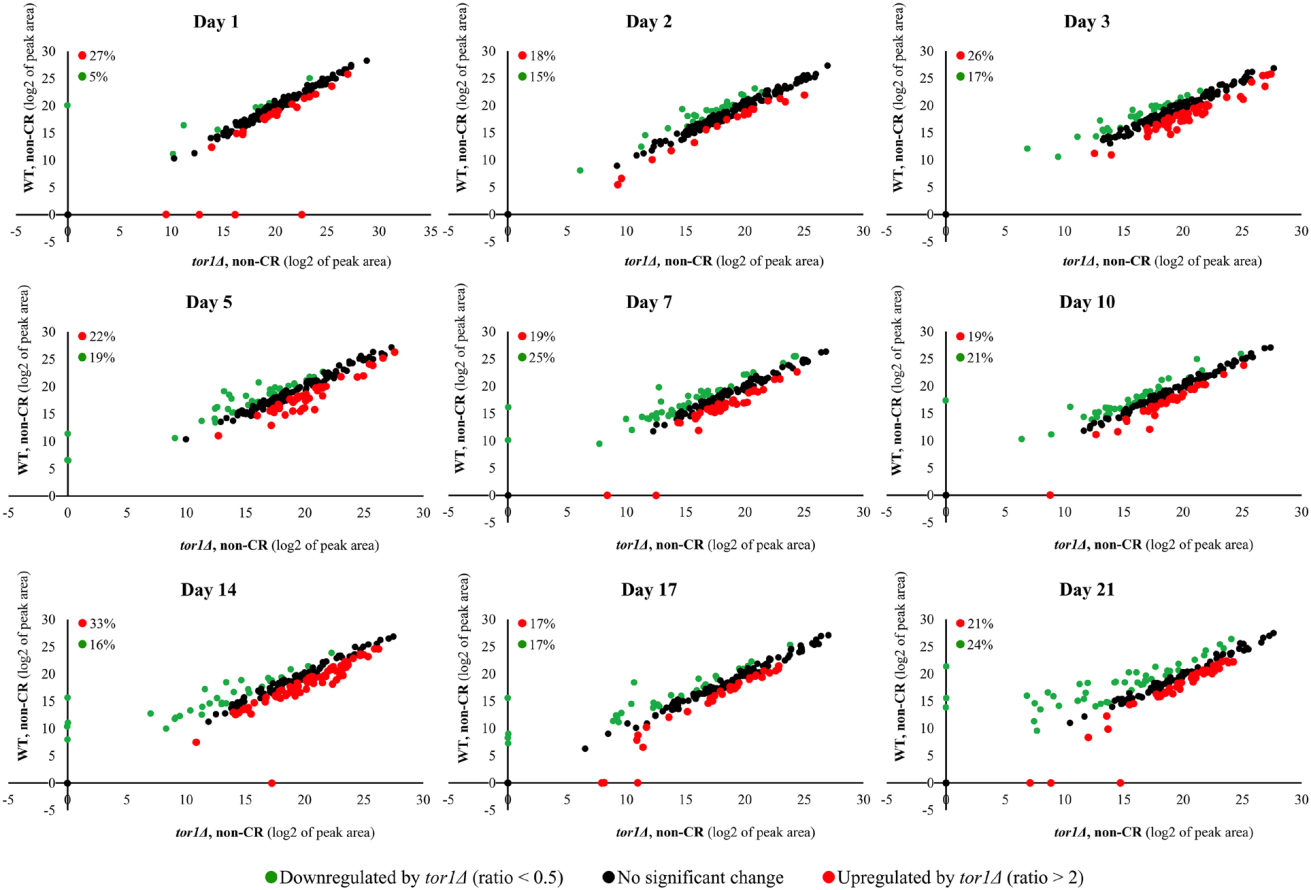
The *tor1Δ* mutation elicits significant changes in the water-soluble metabolome of yeast under non-CR conditions. The WT strain BY4742 and the *tor1*Δ single-gene-deletion mutant strain in the BY4742 genetic background were cultured in the nutrient-rich YP medium initially containing 2% (w/v) glucose (non-CR conditions). Cell aliquots were collected as described in the legend for Figure 2. Metabolic activity quenching, metabolite extraction, and spectrometric identification and quantitation of water-soluble metabolites were carried out as described in Materials and Methods. The data on the relative abundance of water-soluble metabolites within WT and *tor1Δ* cells under non-CR conditions were plotted on a log_2_-log_2_ scale of mass spectrometric peak areas for different metabolites. Each plot provides the percentage abundance of metabolites that were upregulated (ratio > 2; displayed in red) or downregulated (ratio < 0.5; shown in green) by the *tor1Δ* mutation under non-CR conditions of cell culturing.

Akin to CR, the *tor1Δ* mutation significantly altered the water-soluble metabolome of yeast (Figure 3). These *tor1Δ*-driven changes in the spectrum of intracellular water-soluble metabolites were seen on all stages of the chronological aging process in yeast cultured under non-CR conditions (Figure 3). The *tor1Δ* mutation elicited a downregulation of 5% to 25% of all intracellular water-soluble metabolites throughout the entire chronological lifespan (Figure 3). From 17% to 33% of the water-soluble metabolite pool was upregulated by the *tor1Δ* mutation on different days of chronological aging under non-CR conditions (Figure 3).

**Figure 4:**
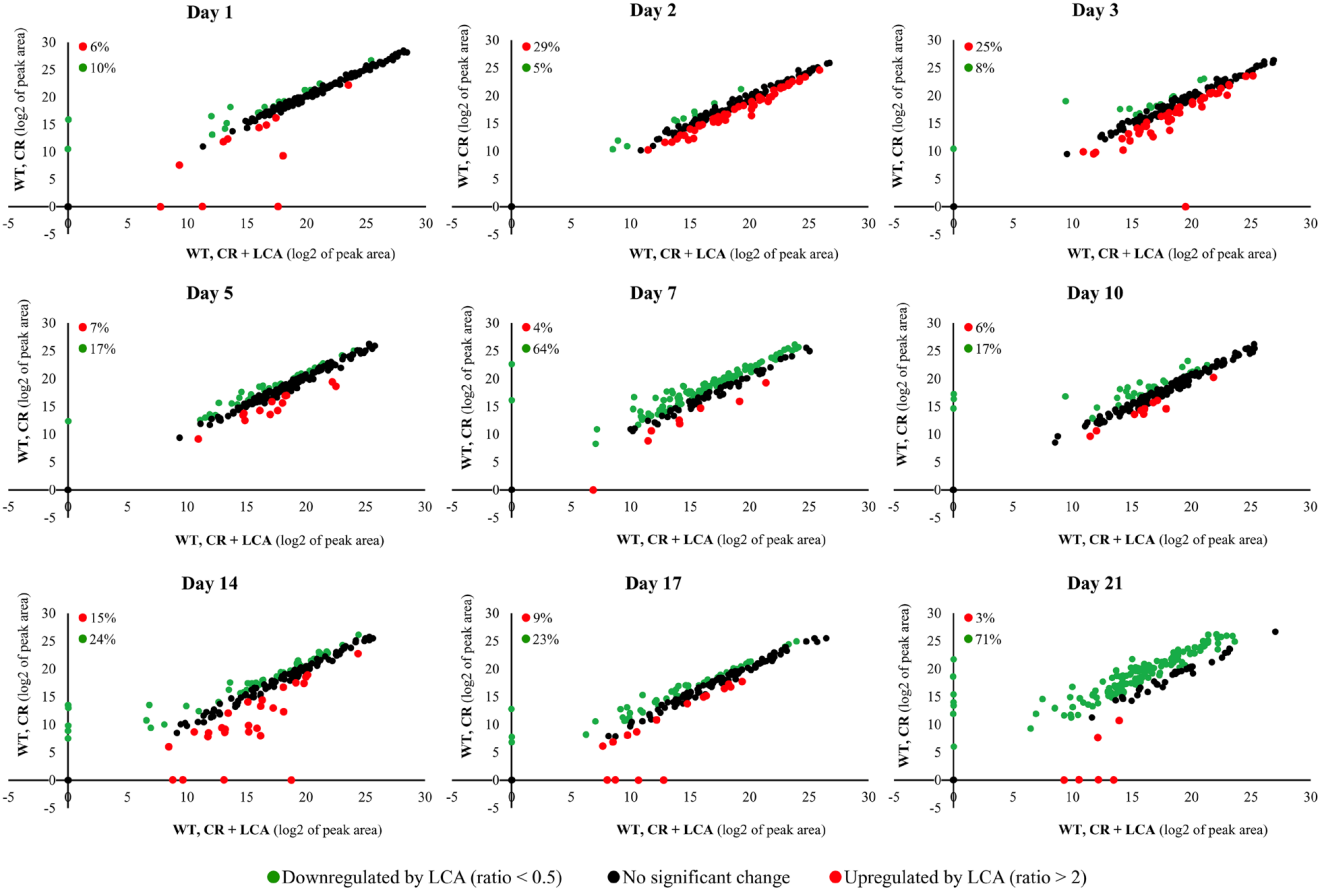
LCA added to calorically restricted WT cells considerably alters their intracellular water-soluble metabolome. The WT strain BY4742 was cultured in the nutrient-rich YP medium initially containing 0.2% (w/v) glucose (CR conditions), with 50 μM LCA or without this bile acid. Cell aliquots were collected as described in the legend for Figure 2. The collected cells were subjected to metabolic activity quenching and metabolite extraction, followed by metabolite identification and quantitation by LC-MS/MS. All these procedures are described in Materials and Methods. Scatter plots for the relative abundance of water-soluble metabolites within WT cells cultured under CR conditions with or without LCA are presented. These plots are on a log_2_-log_2_ scale of mass spectrometric peak areas for different metabolites. The percentage abundance of metabolites that were upregulated (ratio > 2; displayed in red) or downregulated (ratio < 0.5; shown in green) by LCA is provided for each time point.

The intracellular water-soluble metabolome was also considerably changed by LCA (Figure 4). We detected the LCA-driven changes in the water-soluble metabolome on all days following LCA addition to WT cells under CR conditions (Figure 4). An exposure to LCA caused downregulation of 5% to 71% of all intracellular metabolites on various days after LCA addition to calorically restricted WT cells (Figure 4). Some metabolites (from 3% to 29%) were upregulated on different days following LCA addition to these cells (Figure 4).

**Figure 5:**
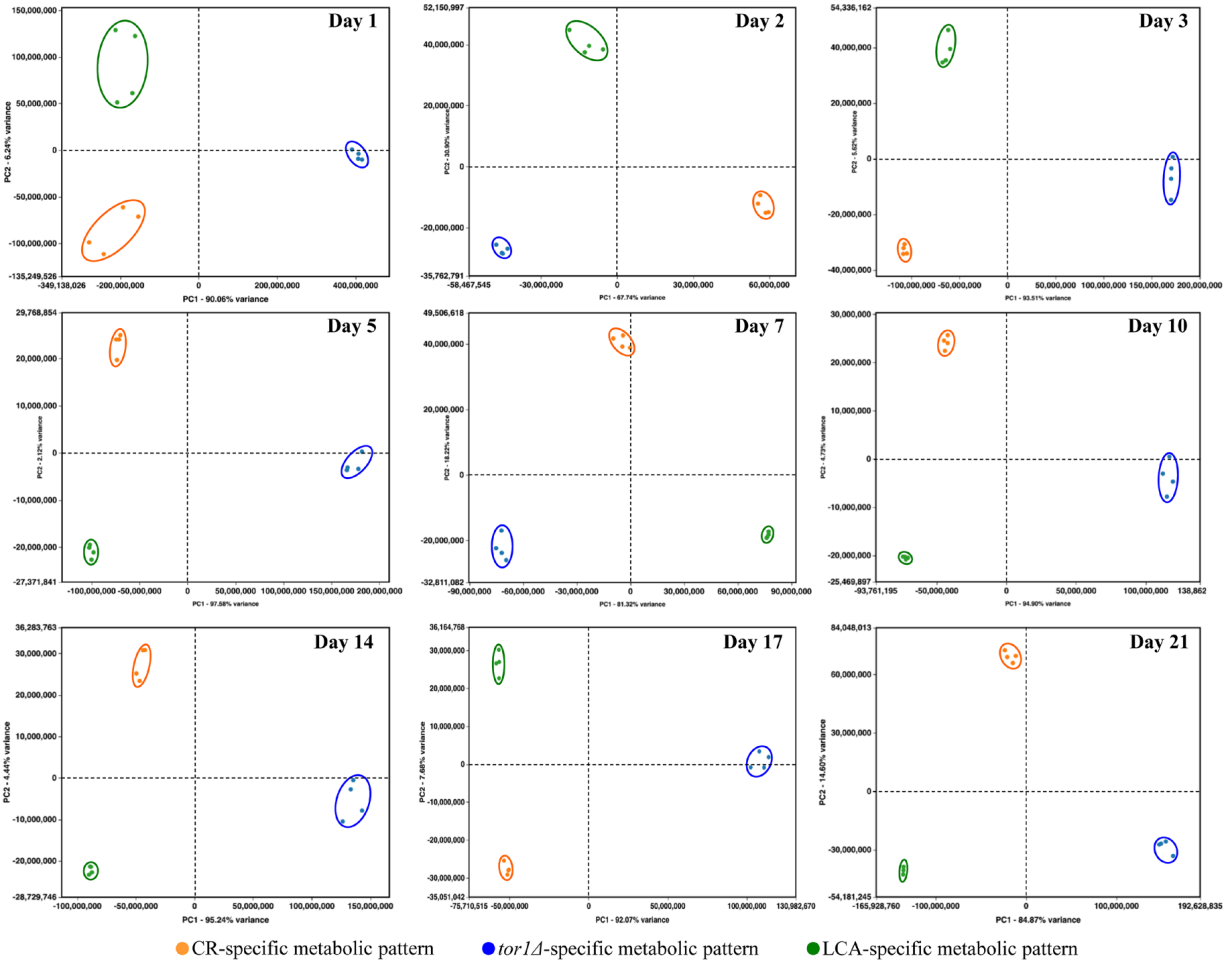
CR, *tor1Δ* and LCA generate different metabolic profiles of 193 water-soluble metabolites throughout the entire chronological lifespan of budding yeast. The WT strain BY4742 was cultured in the nutrient-rich YP medium initially containing 2% (w/v) glucose (non-CR conditions), 0.2% (w/v) glucose (CR conditions) or 0.2% (w/v) glucose and 50 μM LCA (CR + LCA conditions). The *tor1Δ* mutant strain in the BY4742 genetic background was cultured in nutrient-rich YP medium initially containing 2% (w/v) glucose (non-CR conditions). Cell aliquots for metabolic activity quenching and metabolite extraction were collected on days 1, 2, 3, 5, 7, 10, 14, 17 and 21 of culturing. The use of LC-MS/MS to identify and quantitate the intracellular water-soluble metabolites is described in Materials and Methods. A WT strain culture that initially contained 2% (w/v) glucose served as a control non-CR culture for defining the metabolic patterns created by the CR and *tor1Δ* geroprotectors. A WT strain culture that initially contained 0.2% (w/v) glucose without LCA served as a control CR culture for defining the metabolic pattern created by the LCA geroprotector. We normalized the data for all 193 water-soluble metabolites found by log-transforming the data into log_2_ values of mass spectrometric peak areas for different metabolites. Normalized data for all these water-soluble metabolites identified in age-matched cells were used to create the principal component analysis (PCA) plots for comparing the metabolic patterns created by the three different geroprotectors. Data of 2 independent experiments, each being performed twice, are presented.

We normalized the data for the relative concentrations of all 193 water-soluble metabolites identified in age-matched cells cultured under CR conditions, carrying the *tor1Δ* mutation or treated with LCA. The normalization was performed by log-transforming the data into log_2_ values of mass spectrometric peak areas for different metabolites. We used these normalized data to compare the metabolic patterns created by the three different geroprotective interventions at various stages of the chronological aging process. Multivariate analysis of the resulting data set using principal component analysis (PCA) demonstrated that the metabolic profiles of the three geroprotectors are very distinct (Figure 5). We noticed that these three geroprotector-specific metabolic profiles significantly differ from each other in yeast cells recovered on any day of culturing (Figure 5). Indeed, the metabolic patterns specific for CR, *tor1Δ* and LCA were well separated from each other along the PC1 and/or PC2 axes of the PCA plots for various stages of the chronological aging process (Figure 5).

In sum, the above findings indicate that CR, the *tor1Δ* mutation and LCA create different metabolic patterns that remain specific for a particular geroprotector throughout the entire chronological lifespan of *S. cerevisiae*.

### CR creates a unique pattern of the metabolic pathway for sulfur amino acid biosynthesis

**Figure 6:**
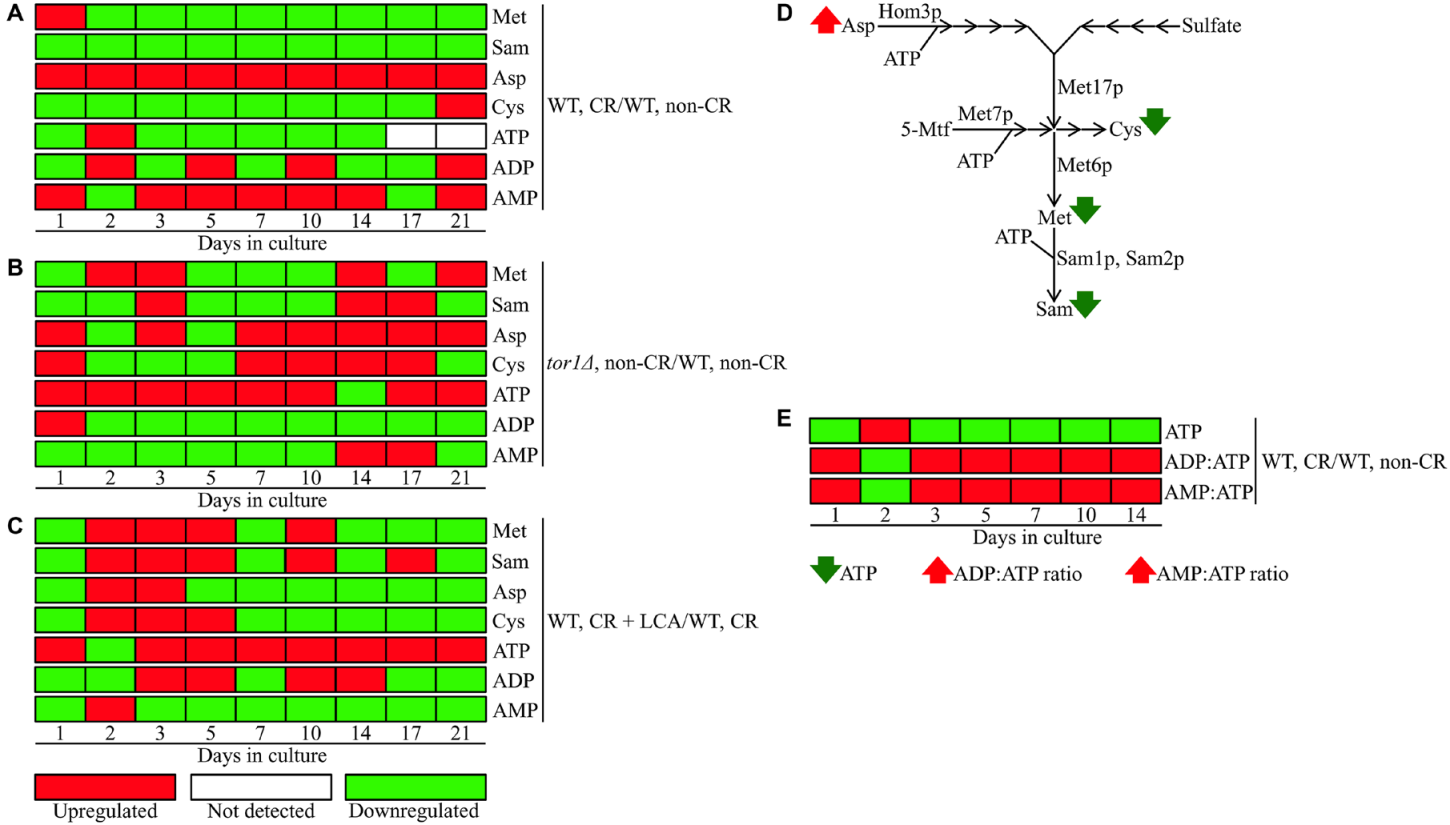
CR, the *tor1Δ* mutation and LCA differently influence the concentrations of methionine (Met), S-adenosylmethionine (Sam), aspartate (Asp), cysteine (Cys) and adenosine phosphate nucleotides throughout the chronological lifespan. The WT and *tor1Δ* mutant strains were cultured, cell aliquots were collected and the metabolomic analysis by LC-MS/MS was performed as described in the legend for Figure 5. The effects of CR (**A**), the *tor1Δ* mutation (**B**) and LCA (**C**) on the relative intracellular concentrations of Met, Sam, Asp, Cys, ATP, ADP and AMP are shown. CR’s effect on the relative intracellular concentration of ATP and the ADP:ATP and AMP:ATP ratios are presented in E. The relative concentrations of metabolites that were significantly upregulated (displayed in red) or downregulated (shown in green) are provided for each time point. ATP was not detected in WT cells recovered on days 17 and 21 of culturing under CR or non-CR conditions without LCA. (**D**) A schematic depiction of how CR affects the metabolic pathway for sulfur amino acid biosynthesis from Asp, sulfate and 5-methyltetrahydrofolate (5-Mtf). (**E**) A graphic representation of changes in the intracellular concentration of ATP and in the ADP:ATP and AMP:ATP ratios in chronologically aging yeast limited in calorie supply. Next to metabolites’ names, arrows denote those whose concentrations or ratios increase (red arrows) or decrease (green arrows) throughout most or all chronological lifespan of yeast cultured under CR conditions. Other abbreviations: Hom3p, aspartate kinase; Met6p, a cobalamin-independent methionine synthase involved (directly or indirectly) in Met and Sam biosynthesis; Met 7p, folylpolyglutamate synthetase indirectly involved in Met, Cys and Sam biosynthesis; Met17p, an *O*-acetyl homoserine-*O*-acetyl serine sulfhydrylase indirectly involved in Met, Cys and Sam biosynthesis; Sam1p and Sam2p, *S*-adenosylmethionine synthetases 1 and 2.

**Figure 7:**
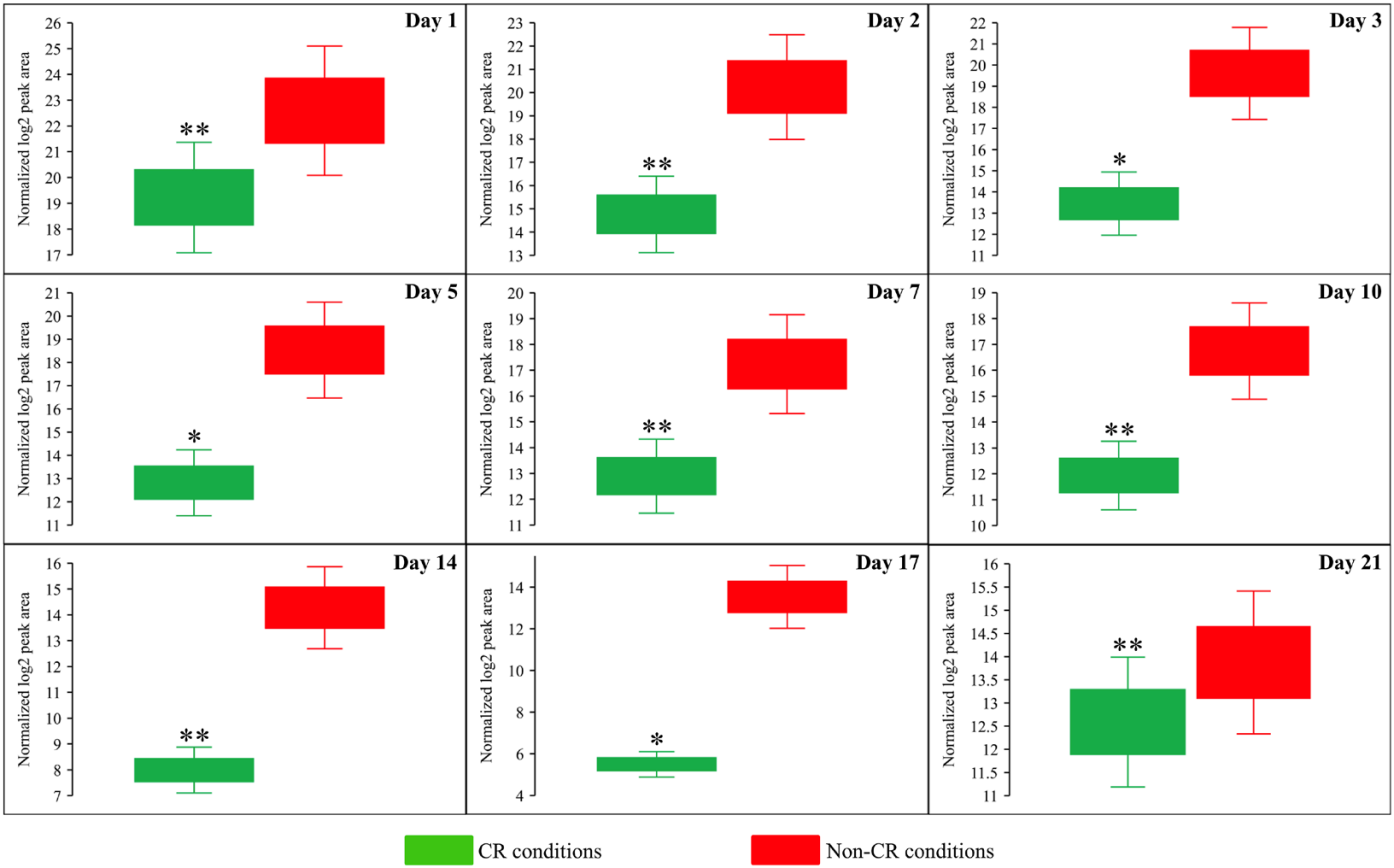
CR significantly decreases the intracellular concentration of *S*-adenosylmethionine (Sam) in yeast cells recovered on any day of culturing. WT strain BY4742 was cultured in the nutrient-rich YP medium initially containing 2% (w/v) glucose (non-CR conditions) or 0.2% (w/v) glucose (CR conditions). Cell aliquots were collected and the metabolomic analysis by LC-MS/MS was performed as described in the legend for Figure 5. The concentrations of Sam within WT cells cultured under CR or non-CR conditions are shown as the normalized log_2_ values of mass spectrometric peak areas for Sam. The *p value*s for comparing the means of two groups were calculated using an unpaired two-tailed *t* test described in Materials and Methods. ^*^ *p* < 0.05, ^**^ *p* < 0.01 and ^***^ *p* < 0.001. Data of 2 independent experiments, each being performed twice, are presented.

We noticed that CR, but not the *tor1Δ* mutation or LCA, significantly lowers the concentration of *S*-adenosylmethionine (Sam) in yeast cells recovered on any day of culturing (Figure 6A–6C and Figure 7). Indeed, the intracellular concentration of Sam was decreased in yeast cultured under CR conditions throughout the entire chronological lifespan (Figure 6A and Figure 7). In contrast, Sam concentrations in yeast cells carrying the *tor1Δ* mutation or treated with LCA fluctuated at various stages of the chronological aging process in a seemingly random manner (Figure 6B and 6C).

**Figure 8:**
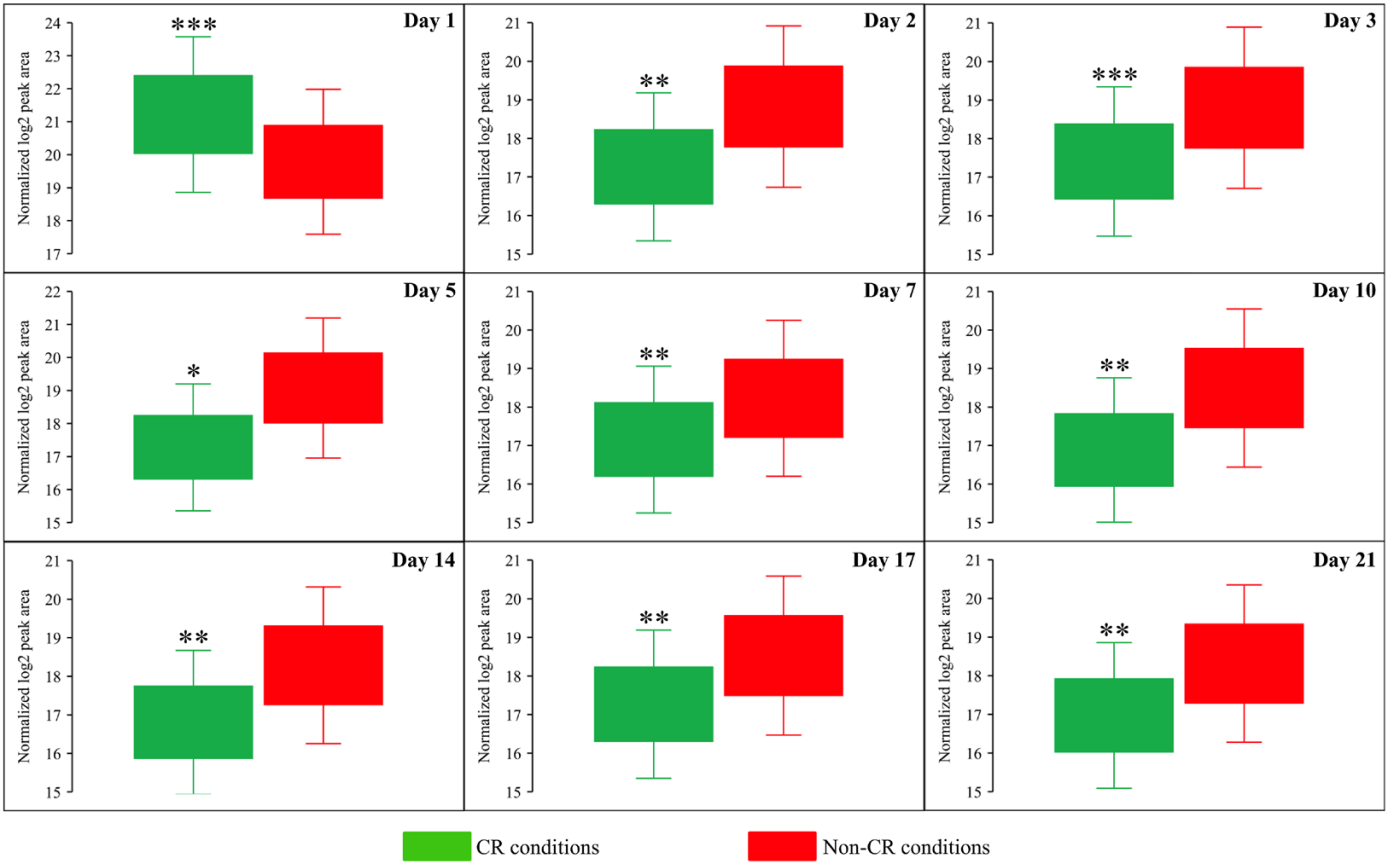
CR significantly decreases the intracellular concentration of methionine (Met) during most days of yeast cell culturing. WT strain BY4742 was cultured in the nutrient-rich YP medium initially containing 2% (w/v) glucose (non-CR conditions) or 0.2% (w/v) glucose (CR conditions). Cell aliquots were collected and the metabolomic analysis by LC-MS/MS was performed as described in the legend for Figure 5. The concentrations of Met within WT cells cultured under CR or non-CR conditions are shown as the normalized log_2_ values of mass spectrometric peak areas for Met. The *p value*s for comparing the means of two groups were calculated using an unpaired two-tailed *t* test described in Materials and Methods. ^*^ *p* < 0.05, ^**^ *p* < 0.01 and ^***^ *p* < 0.001. Data of 2 independent experiments, each being performed twice, are presented.

We also found that beginning of day 2 of culturing, CR causes a significant decline in methionine (Met) concentration within yeast cells at various chronological aging phases (Figure 6A and Figure 8). On the contrary, the *tor1Δ* mutation and LCA elicited alterations in the intracellular concentrations of Met that randomly fluctuated on different days of cell culturing (Figure 6B and 6C).

**Figure 9:**
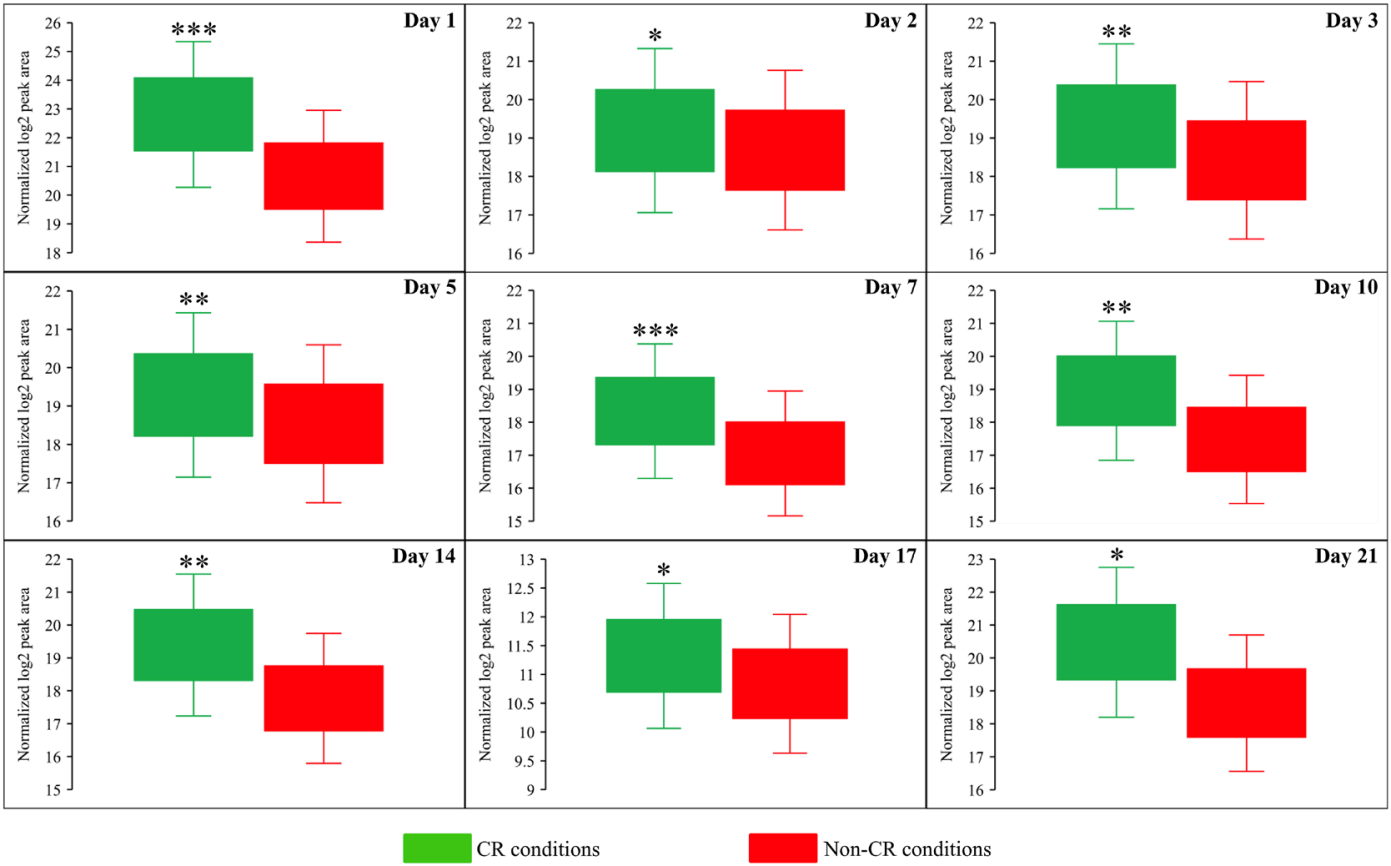
CR significantly increases the intracellular concentration of aspartate (Asp) throughout the entire chronological lifespan of budding yeast. WT strain BY4742 was cultured in the nutrient-rich YP medium initially containing 2% (w/v) glucose (non-CR conditions) or 0.2% (w/v) glucose (CR conditions). Cell aliquots were collected and the metabolomic analysis by LC-MS/MS was performed as described in the legend for Figure 5. The concentrations of Asp within WT cells cultured under CR or non-CR conditions are shown as the normalized log_2_ values of mass spectrometric peak areas for Asp. The *p value*s for comparing the means of two groups were calculated using an unpaired two-tailed *t* test described in Materials and Methods. ^*^ *p* < 0.05, ^**^ *p* < 0.01 and ^***^ *p* < 0.001. Data of 2 independent experiments, each being performed twice, are presented.

**Figure 10:**
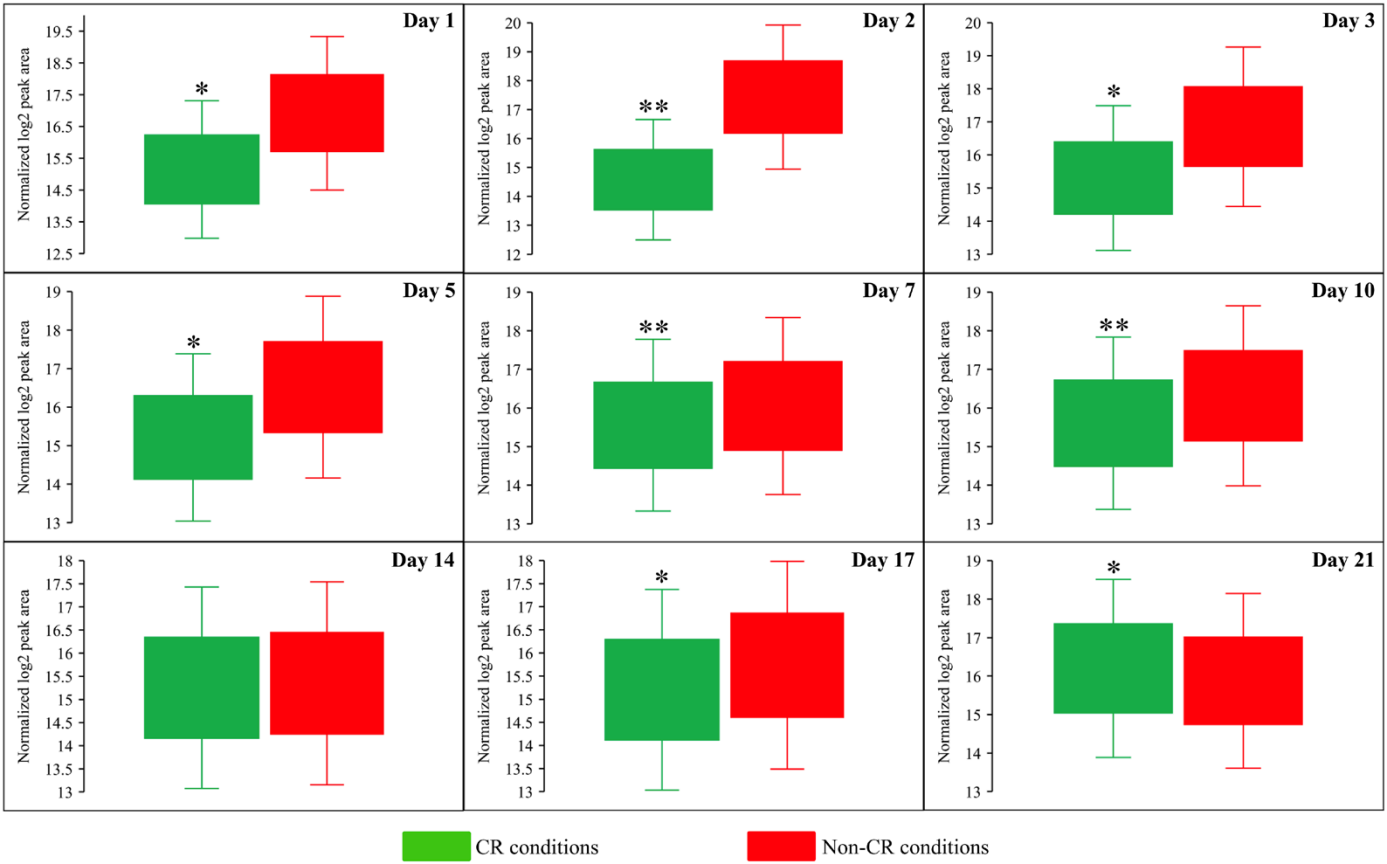
CR significantly decreases the intracellular concentration of cysteine (Cys) during most days of yeast cell culturing. WT strain BY4742 was cultured in the nutrient-rich YP medium initially containing 2% (w/v) glucose (non-CR conditions) or 0.2% (w/v) glucose (CR conditions). Cell aliquots were collected and the metabolomic analysis by LC-MS/MS was performed as described in the legend for Figure 5. The concentrations of Cys within WT cells cultured under CR or non-CR conditions are shown as the normalized log_2_ values of mass spectrometric peak areas for Cys. The *p value*s for comparing the means of two groups were calculated using an unpaired two-tailed *t* test described in Materials and Methods. ^*^ *p* < 0.05, ^**^ *p* < 0.01. Data of 2 independent experiments, each being performed twice, are presented.

Both Sam and Met are the two products of the metabolic pathway for sulfur amino acid biosynthesis from aspartate (Asp), sulfate and 5-methyltetrahydrofolate (5-Mtf) (Figure 6D) [64]. This pathway also leads to the biosynthesis of cysteine (Cys) (Figure 6D) [64]. We found that CR significantly increases the concentration of Asp throughout the entire chronological lifespan of *S. cerevisiae* (Figure 6A and Figure 9). CR exhibited the opposite effect on Cys concentration during most days (other than day 21) of yeast cell culturing (Figure 6A and Figure 10). Akin to the impacts of the *tor1Δ* mutation and LCA on the intracellular concentrations of Sam and Met, these two geroprotectors randomly affected Asp and Cys intracellular concentrations throughout the chronological lifespan (Figure 6B and 6C).

Altogether, the above findings suggest that CR (but not the *tor1Δ* mutation or LCA) regulates the metabolite flow along the metabolic pathway for sulfur amino acid biosynthesis in a specific manner. This geroprotective diet suppresses the biosynthesis of Met, Sam and Cys from Asp, sulfate and 5-Mtf throughout the chronological lifespan (Figure 6D).

**Figure 11:**
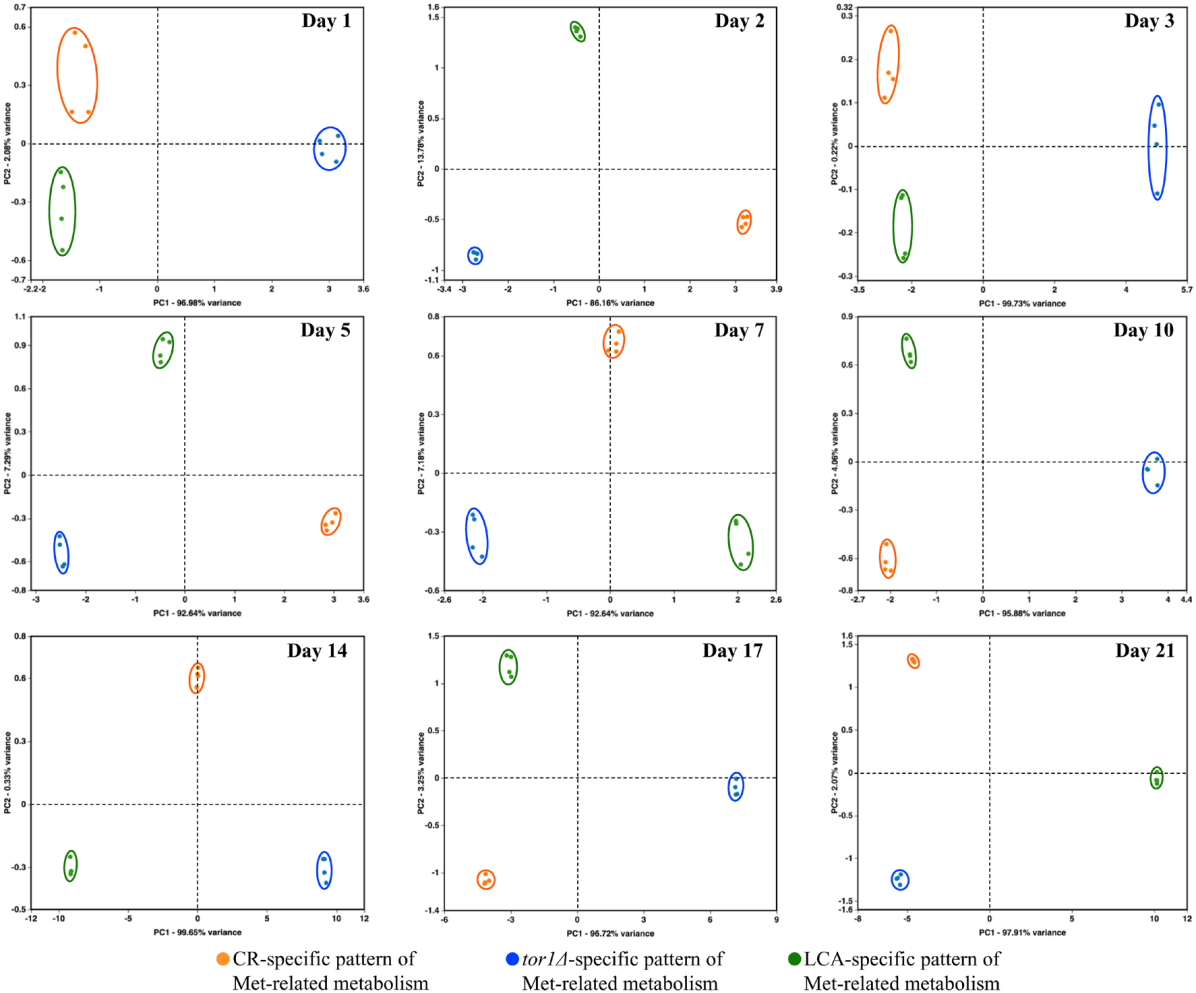
CR, *tor1Δ* and LCA differently affect the metabolic pathway for sulfur amino acid biosynthesis on various days of culturing. The WT and *tor1Δ* mutant strains were cultured, cell aliquots were collected and the metabolomic analysis by LC-MS/MS was performed as described in the legend for Figure 5. A WT strain culture that initially contained 2% (w/v) glucose served as a control non-CR culture for defining the metabolic patterns created by the CR and *tor1Δ* geroprotectors. A WT strain culture that initially contained 0.2% (w/v) glucose without LCA served as a control CR culture for defining the metabolic pattern created by the LCA geroprotector. The data on the relative concentrations of all four metabolites (i.e., Asp, Cys, Met and Sam) within the sulfur amino acid biosynthetic pathway were normalized in age-matched cells. Multivariate analysis by PCA was then used to compare how the CR, *tor1Δ* and LCA geroprotectors affect the metabolic pathway for sulfur amino acid biosynthesis on various days of culturing. Data of 2 independent experiments, each being performed twice, are presented.

We normalized the data on the relative concentrations of all four metabolites (i.e., Asp, Cys, Met and Sam) within the sulfur amino acid biosynthetic pathway. This normalization was performed by log-transforming the data into log_2_ values of mass spectrometric peak areas of the four metabolites for age-matched cells cultured under CR conditions, carrying the *tor1Δ* mutation or treated with LCA. We then applied multivariate analysis by PCA to compare how the three geroprotectors affect the metabolic pathway for sulfur amino acid biosynthesis on various days of culturing. We found that the pathway patterns specific for CR, *tor1Δ* and LCA are well separated from each other along the PC1 and/or PC2 axes of the PCA plots (Figure 11). Notably, these geroprotector-specific patterns were seen at diverse stages of the chronological aging process (Figure 11). We concluded that the three geroprotectors differently influence the sulfur amino acid biosynthetic pathway throughout the chronological lifespan.

### CR significantly increases the intracellular ADP:ATP and AMP:ATP ratios

Three enzymatic reactions of the metabolic pathway for sulfur amino acid biosynthesis from Asp, sulfate and 5-Mtf use ATP as a co-substrate (Figure 6D) [64]. These reactions are catalyzed by Hom3p, Met7p, Sam1p and Sam2p (Figure 6D) [64]. Therefore, we expected that the CR-driven remodeling of this metabolic pathway could contribute to a change of ATP, ADP and AMP concentrations within chronologically aging yeast limited in calorie supply.

We found that CR, unlike the *tor1Δ* mutation or LCA, significantly decreases the intracellular concentration of ATP on any day of culturing other than day 2 (Figure 6A–6C; Supplementary Figure 1). We noticed that ATP is not detected in WT cells recovered on days 17 and 21 of culturing under CR conditions without LCA or under non-CR conditions. Unlike CR, the *tor1Δ* mutation and LCA caused a significant rise in ATP concentration during most cell culturing days other than day 14 and day 2, respectively (Figure 6B and 6C).

We also found that CR significantly alters (i.e., increases or decreases) the intracellular concentration of ADP during most days of yeast culturing (Figure 6A and Supplementary Figure 2). Moreover, CR increased AMP concentration within yeast cells on most chronological lifespan days (Figure 6A and Supplementary Figure 3). Unlike CR, the *tor1Δ* mutation and LCA significantly decreased the intracellular concentrations of ADP and AMP at various consecutive phases of chronological aging (compare Figure 6A to Figure 6B and 6C).

We normalized the data on the concentrations of all three adenosine phosphate nucleotides (i.e., ATP, ADP and AMP) within age-matched cells cultured under CR conditions, carrying the *tor1Δ* mutation or treated with LCA. The normalization was performed by log-transforming the data into log_2_ values of mass spectrometric peak areas for the three adenosine phosphate nucleotides. We used multivariate analysis by PCA to compare the normalized data throughout the chronological lifespan. Yeast recovered on days 17 and 21 were not examined by this PCA because ATP was not detected in WT cells cultured under CR or non-CR conditions without LCA. Our PCA revealed that the ATP, ADP and AMP patterns specific for CR, *tor1Δ* and LCA are well separated from each other along the PC1 and/or PC2 axes of the PCA plots (Supplementary Figure 4). Therefore, we concluded that the three geroprotectors differently affect the intracellular concentrations of adenosine phosphate nucleotides at diverse phases of yeast chronological aging.

**Figure 12:**
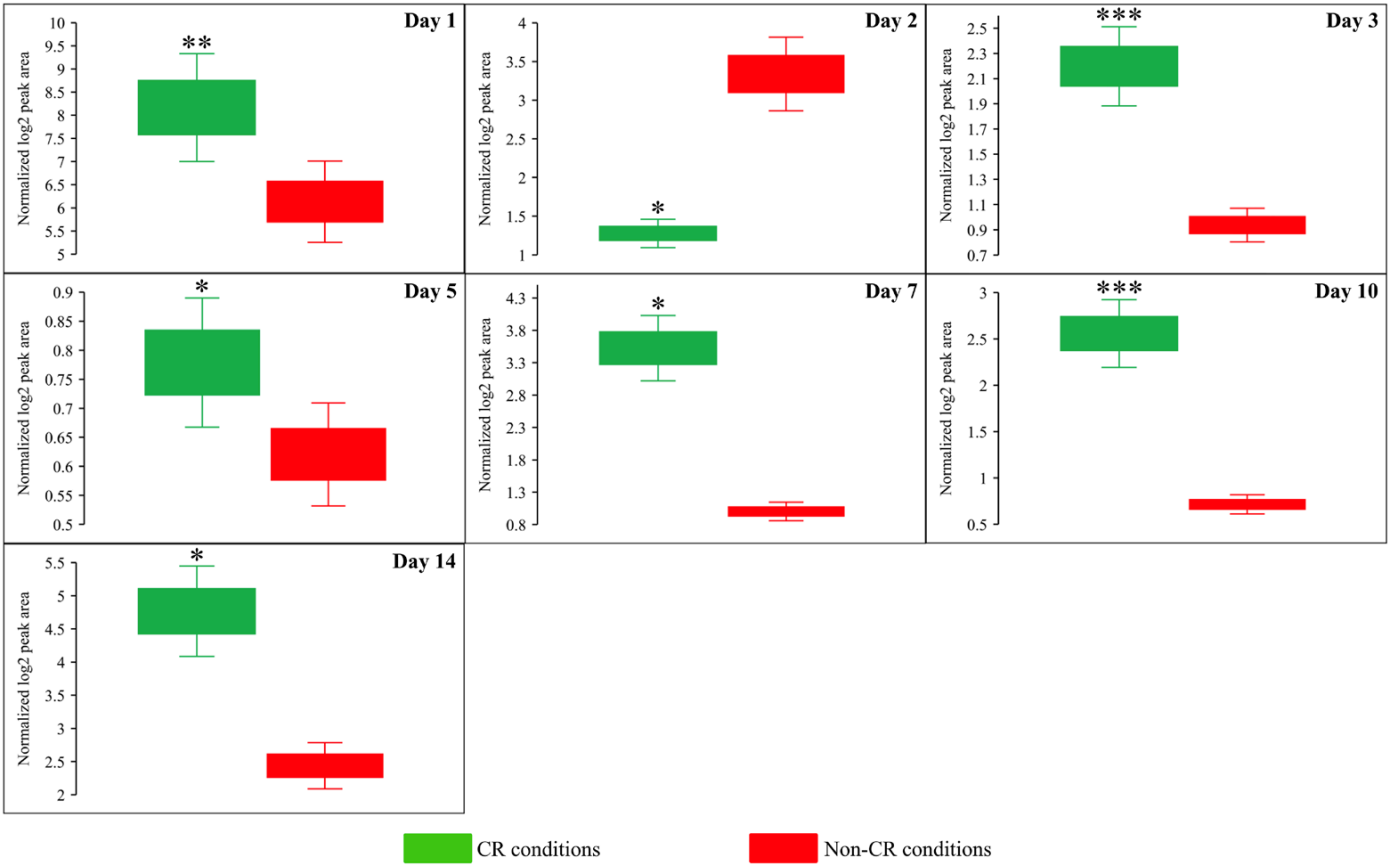
CR significantly increases the AMP:ATP ratios during most days of yeast cell culturing. WT strain BY4742 was cultured in the nutrient-rich YP medium initially containing 2% (w/v) glucose (non-CR conditions) or 0.2% (w/v) glucose (CR conditions). Cell aliquots were collected and the metabolomic analysis by LC-MS/MS was performed as described in the legend for Figure 5. The AMP:ATP ratios within WT cells cultured under CR or non-CR conditions are shown as the normalized log_2_ values of mass spectrometric peak areas for AMP and ATP. The AMP:ATP ratios were not calculated for yeast recovered on days 17 and 21 of culturing because ATP was not detected in WT cells cultured under CR or non-CR conditions without LCA. The *p value*s for comparing the means of two groups were calculated using an unpaired two-tailed *t* test described in Materials and Methods. ^*^ *p* < 0.05, ^**^ *p* < 0.01, ^***^ *p* < 0.001. Data of 2 independent experiments, each being performed twice, are presented.

**Figure 13:**
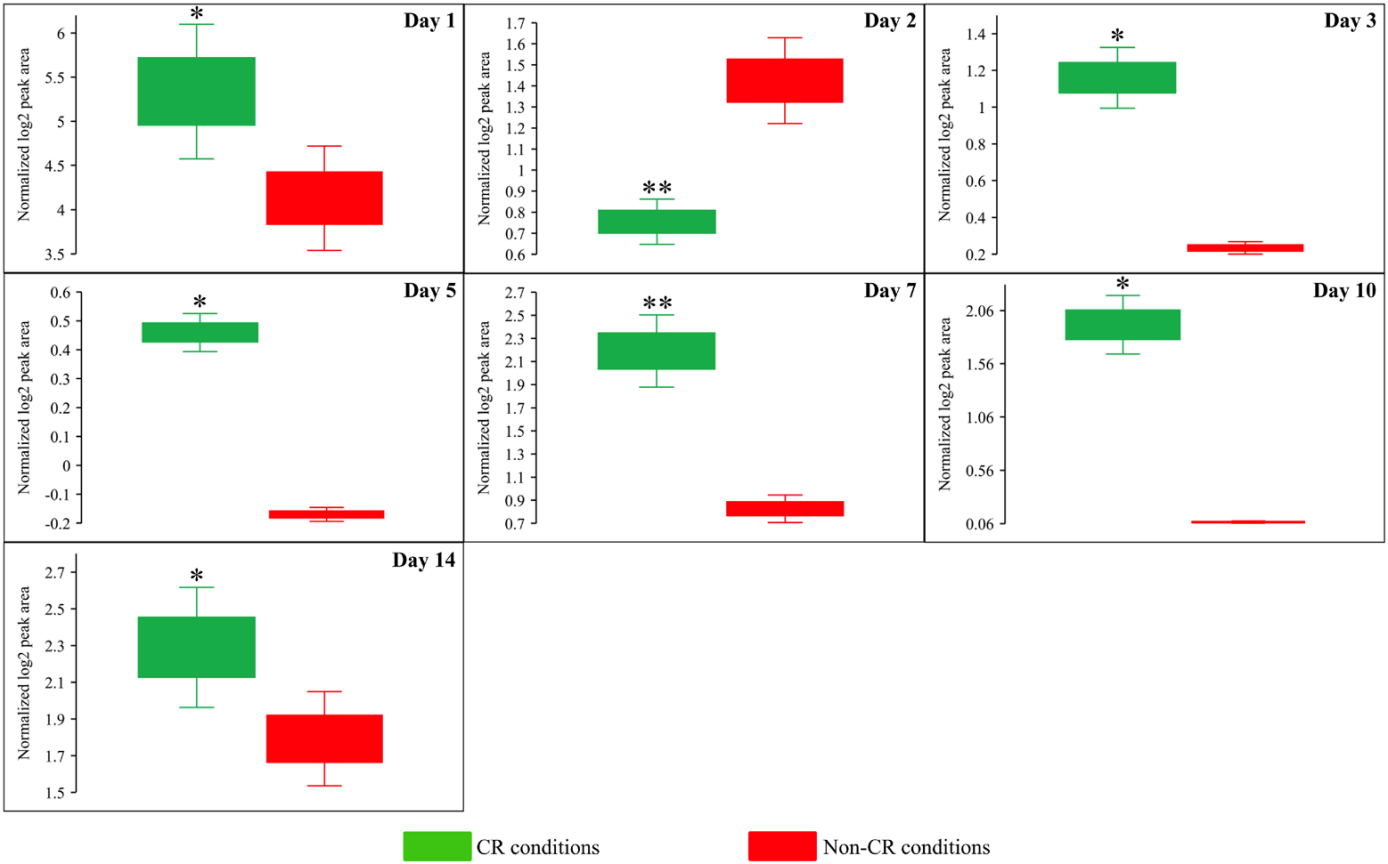
CR significantly increases the ADP:ATP ratios during most days of yeast cell culturing. WT strain BY4742 was cultured in the nutrient-rich YP medium initially containing 2% (w/v) glucose (non-CR conditions) or 0.2% (w/v) glucose (CR conditions). Cell aliquots were collected and the metabolomic analysis by LC-MS/MS was performed as described in the legend for Figure 5. The ADP:ATP ratios within WT cells cultured under CR or non-CR conditions are shown as the normalized log_2_ values of mass spectrometric peak areas for ADP and ATP. The ADP:ATP ratios were not calculated for yeast recovered on days 17 and 21 of culturing because ATP was not detected in WT cells cultured under CR or non-CR conditions without LCA. The *p value*s for comparing the means of two groups were calculated using an unpaired two-tailed *t* test described in Materials and Methods. ^*^ *p* < 0.05, ^**^ *p* < 0.01. Data of 2 independent experiments, each being performed twice, are presented.

Due to the above effects of CR on the relative intracellular concentrations of adenosine phosphate nucleotides, this low-calorie diet significantly increased the ADP:ATP and AMP:ATP ratios during most days of yeast cell culturing (Figure 6E, Figure 12 and Figure 13). We applied multivariate analysis by PCA to compare the ADP:ATP and AMP:ATP ratios specific for CR, *tor1Δ* and LCA. Again, the data for yeast recovered on days 17 and 21 were not subject to this PCA because no ATP was detected in WT cells cultured under CR or non-CR conditions in the absence of LCA. Our PCA revealed that the normalized by log-transformation data for the ADP:ATP and AMP:ATP ratios specific for CR, *tor1Δ* and LCA are well separated from each other along the PC1 and/or PC2 axes of the PCA plots (Supplementary Figure 5). This separation was observed for all days of cell culturing subjected to PCA (Supplementary Figure 5). Based on these findings, we concluded that the effects of the three geroprotectors on the intracellular ADP:ATP and AMP:ATP ratios differ throughout yeast chronological lifespan.

## DISCUSSION

In this study, we used a recently developed LC-MS/MS method of non-targeted metabolomics to investigate how three different geroprotective interventions affect the intracellular water-soluble metabolome of chronologically aging *S. cerevisiae*. These interventions include CR (a robust dietary geroprotector), the *tor1Δ* mutation (an evolutionarily conserved genetic geroprotector) and LCA (a potent pharmacological geroprotector in yeast). Our findings provide the first evidence that the three different geroprotectors create distinct metabolic patterns throughout the budding yeast’s entire chronological lifespan.

It needs to be emphasized that our metabolomic analysis of the geroprotector-delayed chronological aging in yeast compared the intracellular concentrations of 193 structurally and functionally diverse water-soluble metabolites. Supplementary Table 4 provides the names of all these metabolites. AMP, ADP, ATP, FAD^+^, FMN, FADH_2_, NAD^+^, NADH, NADP^+^, NADPH, other nucleotides, amino acids, monosaccharides, intermediates of glycolysis and tricarboxylic cycle intermediates were among these metabolites (Supplementary Table 4). Thus, the observed geroprotector-specific remodeling of the metabolic pattern affects the major pathways of cellular metabolism.

Our study identified a distinct metabolic pattern created by the CR geroprotector. We found two characteristic features that distinguish the CR-specific metabolic pattern from the cellular metabolism patterns created by the *tor1Δ* and LCA geroprotectors. One characteristic feature that distinguishes the CR-specific metabolic design is CR’s ability to suppress the biosynthesis of Met, Sam and Cys from Asp, sulfate and 5-Mtf throughout the chronological lifespan. The other characteristic feature of the CR-specific metabolic pattern is a decline in the intracellular concentration of ATP, a rise in the intracellular concentrations of ADP and AMP, and an increase in the ADP:ATP and AMP:ATP ratios at various consecutive phases of chronological aging.

Importantly, both features of the CR-specific remodeling of cellular metabolism are likely to contribute to yeast chronological aging delay by CR.

Indeed, Met restriction is known to extend longevity in yeast and other evolutionary distant eukaryotes [65–69]. Furthermore, a recent study revealed that the *met6Δ* and *met17Δ* mutations (Figure 6D) decrease the intracellular concentration of Met and extend yeast replicative lifespan (RLS) under non-CR conditions in a nutrient-limited medium [70]. Moreover, the excessive quantities of exogenously added Met abrogate the CR-dependent extension of yeast RLS in a nutrient-limited medium [70]. Several mechanisms of yeast CLS extension by lowering the intracellular concentration of Met, Sam and Cys have been proposed. One likely mechanism is that a decline in intracellular Met concentration lowers the Met-dependent activation of the pro-aging Tor1 (target of rapamycin complex 1) pathway, thereby suppressing the inhibitory effect of Tor1 on the anti-aging process of autophagy [71]. Another possible mechanism is that a deterioration in the intracellular concentration of Met activates the anti-aging process for proteasomal degradation of damaged and dysfunctional proteins [70]. Furthermore, a decrease in intracellular Sam concentration can weaken the pro-aging Tor1 pathway because it suppresses the protein phosphatase Ppa2p (inorganic pyrophosphatase 2; a known upstream activator of Tor1) [72]. Moreover, a decline in the intracellular concentrations of Met and Cys can slow the pro-aging process of protein synthesis because these two sulfur amino acids promote a thiolation-driven tRNA activation [73].

The observed CR-specific remodeling of adenosine phosphate nucleotide metabolism is also a likely contributor to yeast chronological aging delay by CR. Indeed, the increases in the intracellular ADP:ATP and AMP:ATP ratios are known to correlate with the activating phosphorylation (or inversely correlate with inactivating dephosphorylation) of the anti-aging heterotrimeric protein complex Snf1 (sucrose non-fermenting complex 1) [74]. These *in vivo* effects of the ADP:ATP and AMP:ATP ratios are not due to AMP or ATP binding to Snf1 [75, 76]. It is feasible that the ADP:ATP and AMP:ATP ratios regulate Snf1 indirectly, either by promoting the activating phosphorylation of Snf1 by the protein kinases Sak1, Tos3 and Elm1 or by suppressing the inactivating dephosphorylation of Snf1 by the protein phosphatase Glc7 [77]. An adenosine phosphate nucleotide can also regulate Snf1 directly. Indeed, ADP binding to Snf1 is known to protect it from inactivating dephosphorylation [75, 76]. As a member of the family of AMP-activated protein kinases [78], Snf1 is a key energy-sensing regulator that phosphorylates and activates or inactivates several protein targets known for their essential roles in defining yeast CLS [77–80].

**Figure 14:**
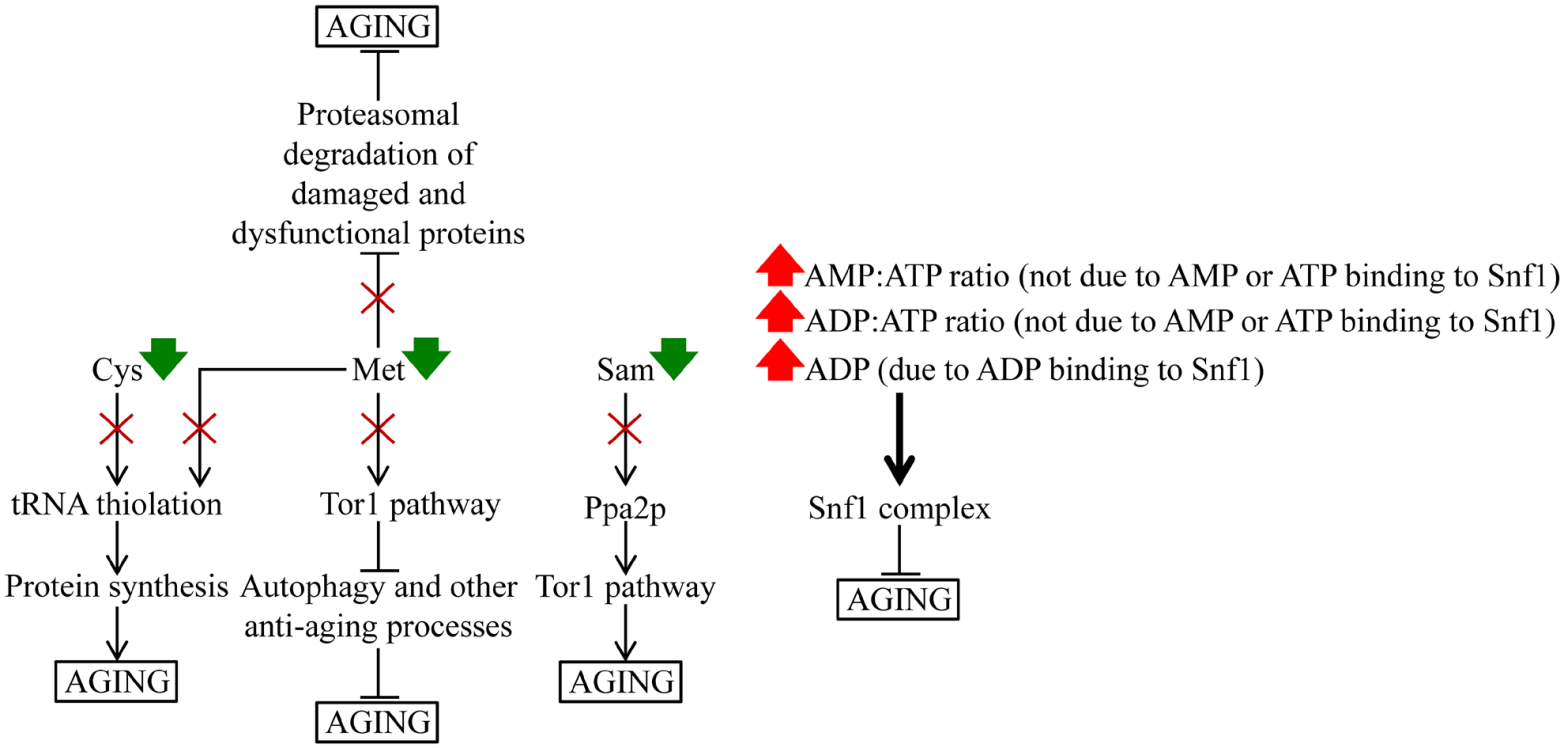
A hypothetical model of how a specific remodeling of cellular metabolism by CR slows down yeast chronological aging. See the text for more details. Abbreviations: Cys, cysteine; Met, methionine; Ppa2p, inorganic pyrophosphatase 2; Sam, *S*-adenosylmethionine; Snf1, sucrose non-fermenting complex 1; Tor1, target of rapamycin complex 1.

These findings suggest a hypothetical model of how the observed CR-specific remodeling of cellular metabolism delays the chronological aging of yeast cultured in the nutrient-rich YP medium. The model is schematically depicted in Figure 14. The key aspects of this model are as follows: 1) a life-long decline in the intracellular concentrations of Cys and Met weakens tRNA thiolation, thus slowing down the pro-aging process of protein synthesis, 2) a decrease of intracellular Met throughout the chronological lifespan attenuates a direct Met-driven stimulation of the pro-aging Tor1 pathway, thereby lowering the inhibitory effect of Tor1 on autophagy and other anti-aging processes, 3) a deterioration in intracellular Met concentration at diverse stages of chronological aging also weakens a Met-dependent suppression of the proteasomal degradation of damaged and dysfunctional proteins, a known anti-aging process, 4) a decline in Sam concentration throughout the chronological lifespan lowers the ability of the protein phosphatase Ppa2p to stimulate the pro-aging Tor1 pathway, and 5) a rise in the ADP:ATP and AMP:ATP ratios on most days of yeast chronological lifespan indirectly (i.e., independent of AMP or ATP binding to Snf1) stimulates the anti-aging protein kinase complex Snf1; Snf1 can also be activated directly, via an ADP binding-dependent protection of Snf1 from inactivating dephosphorylation.

This study created two important questions. First, what is the mechanism responsible for ATP decline under CR conditions? We hypothesize that CR might affect the transcription and/or translation of enzymes involved in ATP synthesis and/or degradation in the cytosol, mitochondria or other cellular locations. Of note, a transcription/translation-based mechanism of suppressing methionine biosynthetic enzymes and transporters underlies the ability of CR to lower intracellular methionine and extend yeast RLS [70]. Second, what are the metabolic changes underlying the extremely efficient longevity extension in yeast culture under CR conditions with LCA? Our findings indicate that LCA applied under CR conditions “overrides” the CR-specific metabolic profile of aging delay. We still do not understand the nature of such LCA-specific overriding. Nonetheless, this observation further supports the notion that metabolism is an essential contributor to eukaryotes’ aging and longevity.

The challenge for the future is to test the model shown in Figure 14. Our ongoing studies address this challenge. In the future, it would also be interesting to define the metabolic “signatures” specific for other geroprotectors, including the *tor1Δ* mutation and LCA.

## MATERIALS AND METHODS

### Yeast strains, media and growth conditions

The wild-type (WT) strain *Saccharomyces cerevisiae* BY4742 (*MATα his3Δ1 leu2Δ0 lys2Δ0 ura3Δ0*) from Thermo Scientific/Open Biosystems was grown in YP medium (1% yeast extract, 2% peptone, both from Fisher Scientific) initially containing the following: 1) 2% (w/v) glucose (Fisher Scientific) as a carbon source, 2) 0.2% (w/v) glucose as a carbon source or 3) 0.2% (w/v) glucose as a carbon source and 50 μM lithocholic acid (LCA). The *tor1Δ* single-gene-deletion mutant strain in the BY4742 genetic background from Thermo Scientific/Open Biosystems was grown in YP medium initially containing 2% (w/v) glucose as a carbon source. Cells were cultured at 30^o^C with rotational shaking at 200 rpm in Erlenmeyer flasks at a “flask volume/medium volume” ratio of 5:1.

### Cell quenching for metabolite extraction

Cell aliquots for metabolite extraction were collected on days 1, 2, 3, 5, 7, 10, 14, 17 and 21 of culturing. After measuring the cell titer with the help of a hemocytometer, a volume of the culture that contains the total number of 5.0 × 10^8^ cells was transferred into a pre-cooled at –5°C 500-ml centrifuge bottle for a Beckman Coulter centrifuge. The centrifuge bottle containing the cells was filled up to the volume of 200 ml with a quenching solution (60% methanol in 155 mM ammonium bicarbonate (ABC) buffer, pH = 8.0) stored at –20°C; methanol of a liquid chromatography-mass spectrometry (LC-MS) grade was used. The bottle was centrifuged in a high-speed Beckman Coulter centrifuge at 11,325 × g for 3 min at –5°C. The bottle was quickly and tenderly recovered from the centrifuge. The bottle’s lid was gently unscrewed, and the supernatant was removed without disturbing the pellet of quenched cells. The cell pellet in each tube was resuspended in 10 ml of ice-cold ABC buffer. The suspension was transferred into a 15-ml high-speed glass centrifuge tube with a polytetrafluoroethylene-lined cap (PYREX) for metabolite extraction. Quenched cells were collected by centrifugation in a clinical centrifuge at 3,000 × g for 3 min at 0°C. The supernatant was promptly removed. The tube with the pelleted quenched cells was kept on dry ice or stored at –80°C until metabolite extraction.

### Metabolite extraction from quenched cells

The following was added to the pelleted quenched cells kept on dry ice or stored at –80°C tube: 1) 2 ml of chloroform stored at –20°C, 2) 1 ml of methanol held at –20°C, 3) 1 ml of ice-cold nanopure water and 4) 200 μl of 425–600 μm acid-washed glass beads (Sigma-Aldrich); LC-MS grade chloroform, methanol and nanopure water were used. The tube’s mouth was covered with aluminum foil. The tube was placed in a foam tube holder kit with a retainer (Thermo Scientific) and vortexed for 30 min at 4°C to facilitate metabolite extraction. The tube was then incubated for 15 min on ice to promote protein precipitation and the separation of the upper aqueous from the lower organic phase. The tube was centrifuged in a clinical centrifuge at 3,000 × g for 10 min at 4°C to separate the following three phases efficiently: the upper aqueous phase (which contained water-soluble metabolites), middle phase (which had cell debris and proteins) and lower organic phase (which included mostly lipids). A glass micropipette was used to transfer the upper aqueous phase (whose total volume was ~ 400 μl) to a 1.5-ml Eppendorf tube containing 800 μl of acetonitrile (ACN; Thermo Scientific) stored at –20°C. The tube was then centrifuged in a tabletop centrifuge at 13,400 **×** g for 10 min at 4°C. The upper portion of a liquid in the tube (a total volume of this portion was 800 μl) was transferred to an MS vial. This vial was stored at 0°C until we used LC-MS/MS to analyze the sample.

### LC-MS/MS analysis of extracted metabolites

LC-MS/MS data were acquired using an Agilent 1100 HPLC system (Agilent Technologies, CA, USA) interfaced with an LTQ Orbitrap Velos mass spectrometer (Thermo Fisher Scientific, Waltham, MA, USA).

Before the sample of extracted metabolites stored in an MS vial at 0°C was analyzed by LC, it was initially subjected to ultrasonic sonication for 15 min. The MS vial with the sample was then vortexed three times (each time for 10 sec) at room temperature. LC separation was carried out using a ZIC-pHILIC column (Merck SeQuant, Umeå, Sweden; 150 × 2.1 mm, 5 μm particle size). Solvent A (a 95:5 (v/v) mixture of nanopure water with ACN (respectively) containing 20 mM ammonium acetate) and solvent B (ACN) were used as the mobile phase for LC; LC-MS grade nanopure water and ACN were used. Supplementary Table 1 provides the LC gradient program used for the chromatographic separation of extracted metabolites. The chromatography column was maintained at 45°C, and the samples were kept at 0°C during metabolite separation.

Mass spectrometric analyses of water-soluble metabolites separated by LC were performed with a Thermo Orbitrap Velos mass spectrometer equipped with a heated electrospray ionization ion source (Thermo Scientific). The mass spectrometer’s analyzer was used for primary ions (MS1). Secondary ions (MS2) were identified with the mass spectrometer’s detector. Supplementary Tables 2 and 3 show the settings used for the data-dependent acquisition of MS1 and MS2 ions, respectively. We used a sample volume of 10 μl for the injection in both the positive and negative ionization modes.

### Metabolite identification and quantitation using the LC-MS/MS raw data

The extracted water-soluble metabolites were identified and quantified with the Compound Discoverer 3.1 software (Fisher Scientific). This software processes raw LC-MS/MS files; it uses MS1 for metabolite quantitation and MS2 for metabolite identification. We used a freely available online library of databases and spectra (https://www.mzcloud.org) to search for MS2 spectra of the raw data.

### Miscellaneous procedures

Statistical analysis was performed using Microsoft Excel’s Analysis ToolPack-VBA. All data on cell survival are presented as mean ± SEM. The *p value*s for comparing the means of two groups using an unpaired two-tailed *t-test* were calculated with the GraphPad Prism 7 statistics software. The logrank test for comparing each pair of survival curves was performed with GraphPad Prism 7. Two survival curves were considered statistically different if the *p value* was less than 0.05. The BioVinci software (https://vinci.bioturing.com/) was used to generate the 2-D plots by PCA of the metabolomic data.

## SUPPLEMENTARY MATERIALS





## Abbreviations

ABC: ammonium bicarbonate
ACN: acetonitrile
CLS: chronological lifespan
CR: caloric restriction
LCA: lithocholic acid
LC: liquid chromatography
MS: mass spectrometry
MS1: primary ion in tandem mass spectrometry
MS2: secondary ion in tandem mass spectrometry
PCA: principal component analysis
Sam: *S*-adenosylmethionine
WT: wild type
5-Mtf: 5-methyltetrahydrofolate
