# Supplementary material for: Caloric restriction creates a metabolic pattern of chronological aging delay that in budding yeast differs from the metabolic design established by two other geroprotectors
Source: Oncotarget. 2021 Mar 30;12(7):608–25. doi: 10.18632/oncotarget.27926 (PMC8021023; doi:10.18632/oncotarget.27926)
Supplement: Supplementary file 1 [file oncotarget-12-608-s001.pdf]

Caloric restriction creates a metabolic pattern of chronological aging delay that in budding yeast differs from the metabolic design established by two other geroprotectors

SUPPLEMENTARY MATERIALS

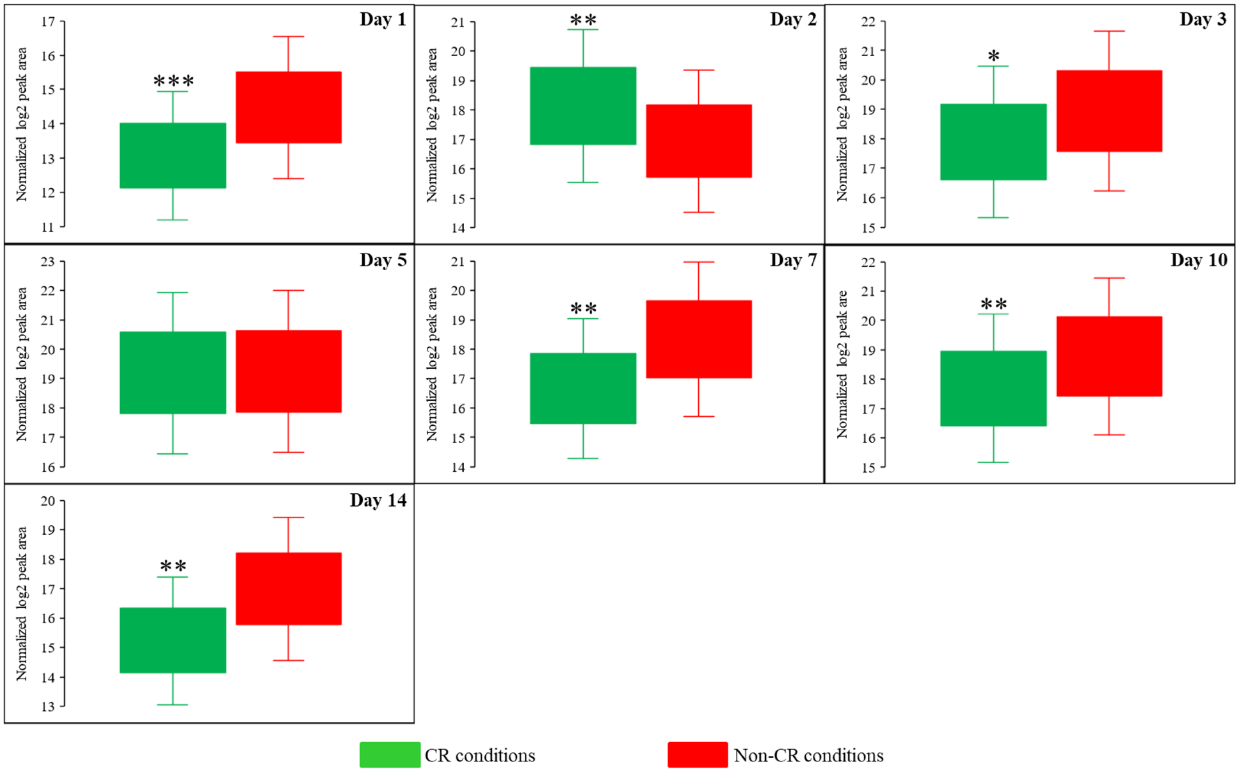

Supplementary Figure 1: CR significantly decreases the intracellular concentration of ATP during most days of yeast cell culturing. WT strain BY4742 was cultured in the nutrient-rich YP medium initially containing 2% (w/v) glucose (non-CR conditions) or 0.2% (w/v) glucose (CR conditions). Cell aliquots were collected and the metabolomic analysis by LC-MS/MS was performed as described in the legend to Figure 5. ATP concentrations within WT cells cultured under CR or non-CR conditions are shown as the normalized $\log_2$ values of mass spectrometric peak areas for ATP. No ATP was detected in WT cells recovered on days 17 and 21 of culturing under CR or non-CR conditions. The p values for comparing the means of two groups were calculated using an unpaired two-tailed t test described in Materials and Methods. $^{*}p < 0.05$, $^{**}p < 0.01$ and $^{***}p < 0.001$. Data of 2 independent experiments, each being performed twice, are presented.

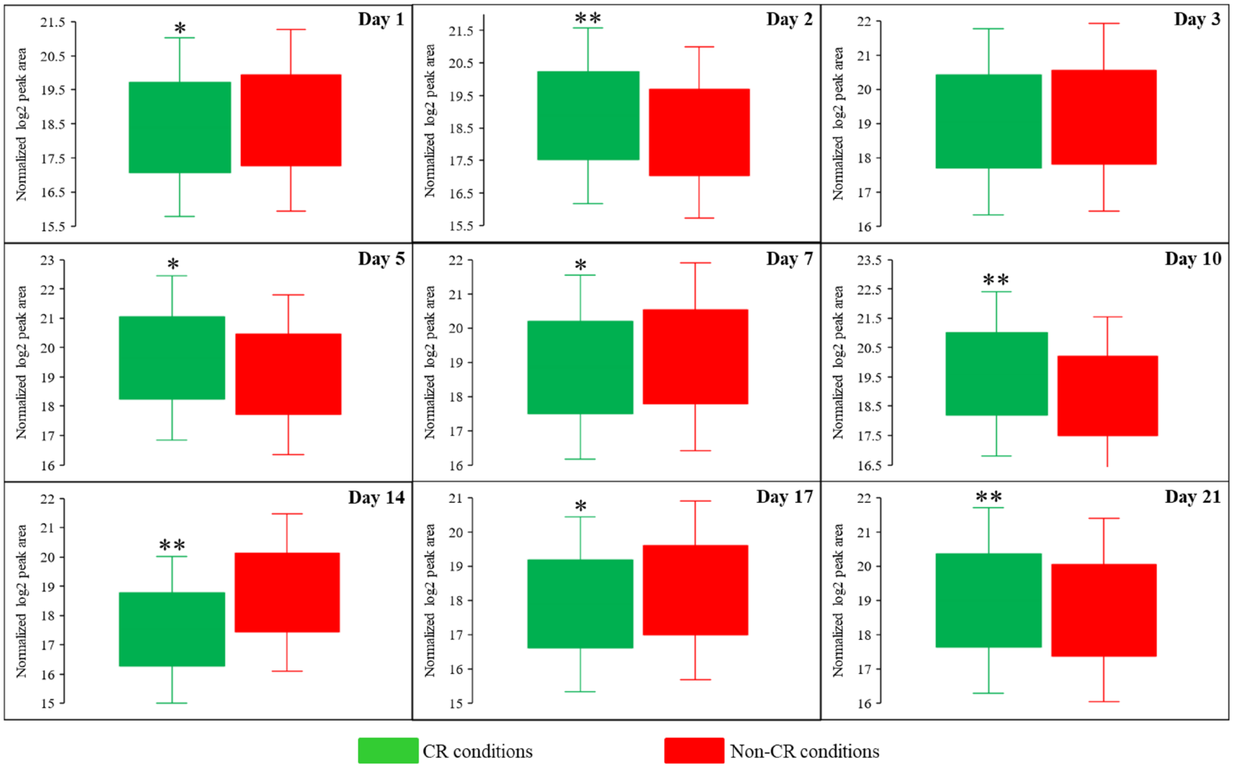

Supplementary Figure 2: CR significantly alters the intracellular concentration of ADP during most days of yeast cell culturing. WT strain BY4742 was cultured in the nutrient-rich YP medium initially containing 2% (w/v) glucose (non-CR conditions) or 0.2% (w/v) glucose (CR conditions). Cell aliquots for metabolic activity quenching and metabolite extraction were collected on days 1, 2, 3, 5, 7, 10, 14, 17 and 21 of culturing. The use of LC-MS/MS to identify and quantitate the intracellular water-soluble metabolites is described in Materials and Methods. ADP concentrations within WT cells cultured under CR or non-CR conditions are shown as the normalized $\log_2$ values of mass spectrometric peak areas for ADP. The p values for comparing the means of two groups were calculated using an unpaired two-tailed t test described in Materials and Methods. $^{*}p < 0.05$ and $^{**}p < 0.01$. Data of 2 independent experiments, each being performed twice, are presented.

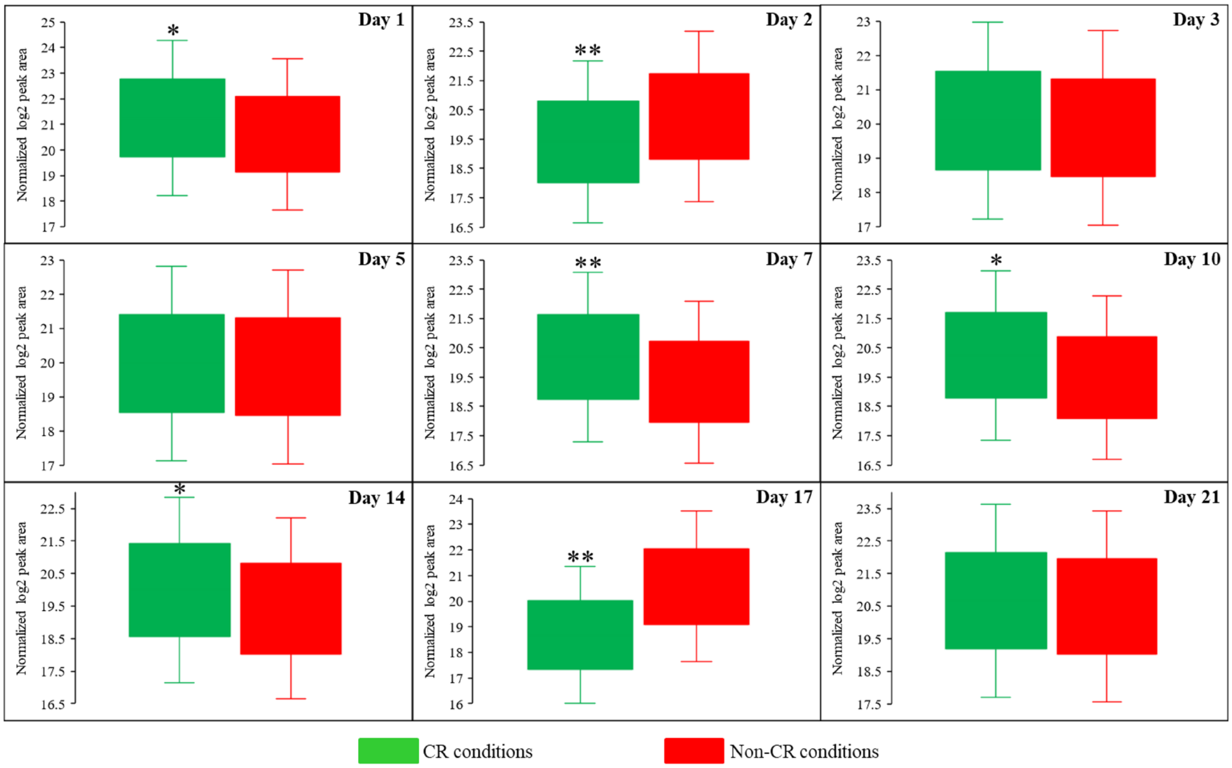

Supplementary Figure 3: CR increases the intracellular concentration of AMP during most days of yeast cell culturing. WT strain BY4742 was cultured in the nutrient-rich YP medium initially containing 2% (w/v) glucose (non-CR conditions) or 0.2% (w/v) glucose (CR conditions). Cell aliquots for metabolic activity quenching and metabolite extraction were collected on days 1, 2, 3, 5, 7, 10, 14, 17 and 21 of culturing. The use of LC-MS/MS to identify and quantitate the intracellular water-soluble metabolites is described in Materials and Methods. AMP concentrations within WT cells cultured under CR or non-CR conditions are shown as the normalized $\log_2$ values of mass spectrometric peak areas for AMP. The p values for comparing the means of two groups were calculated using an unpaired two-tailed t test described in Materials and Methods. $^{*}p < 0.05$ and $^{**}p < 0.01$. Data of 2 independent experiments, each being performed twice, are presented.

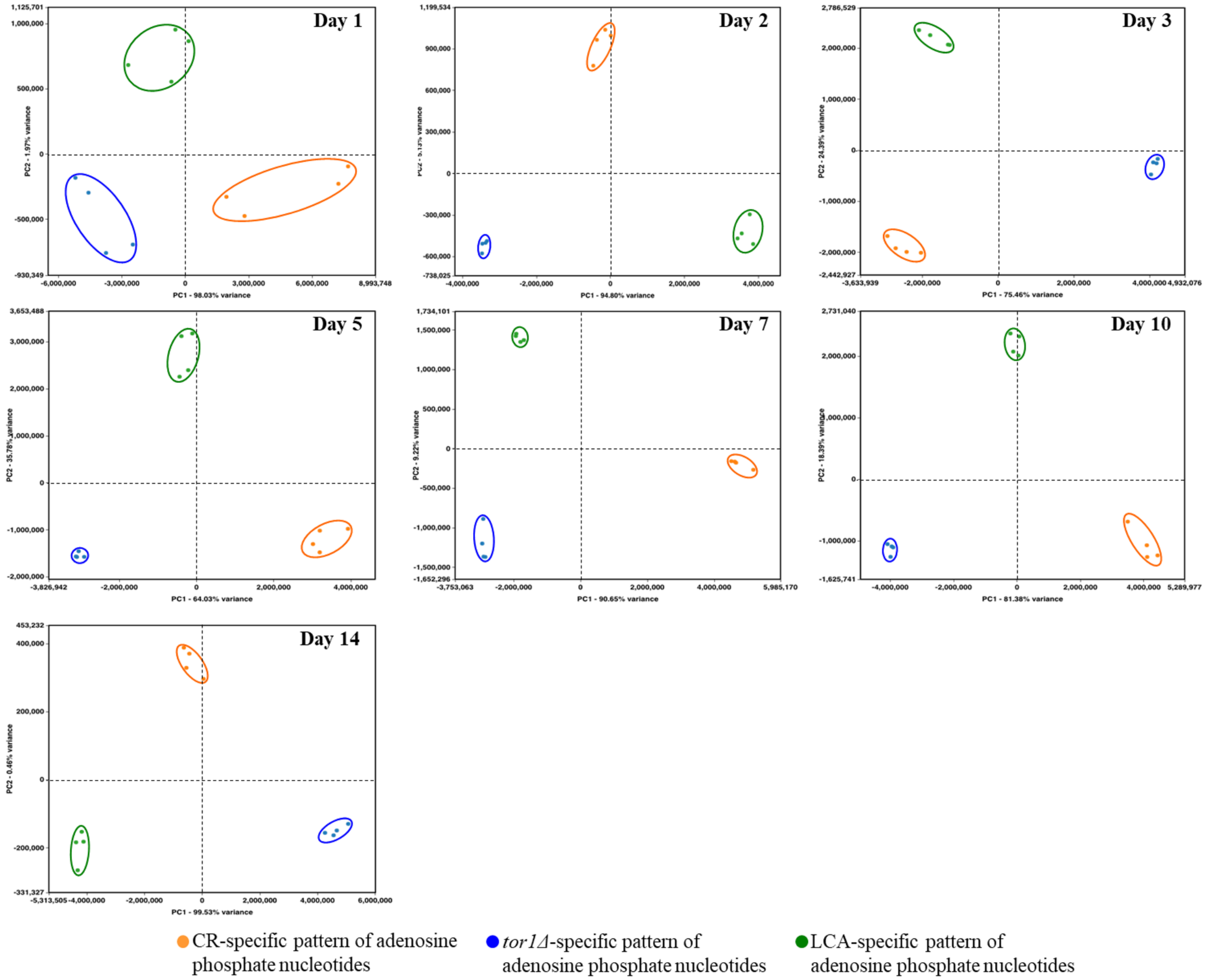

Supplementary Figure 4: CR, *tor1Δ* and LCA differently affect the intracellular concentrations of adenosine phosphate nucleotides (i.e., ATP, ADP and AMP) at diverse phases of yeast chronological aging. The WT strain BY4742 was cultured in the nutrient-rich YP medium initially containing 2% (w/v) glucose (non-CR conditions), 0.2% (w/v) glucose (CR conditions) or 0.2% (w/v) glucose and 50 μM LCA (CR + LCA conditions). The *tor1Δ* mutant strain in the BY4742 genetic background was cultured in nutrient-rich YP medium initially containing 2% (w/v) glucose (non-CR conditions). Cell aliquots for metabolic activity quenching and metabolite extraction were collected on days 1, 2, 3, 5, 7, 10, 14, 17 and 21 of culturing. The use of LC-MS/MS to identify and quantitate the intracellular water-soluble metabolites is described in Materials and Methods. A WT strain culture that initially contained 2% (w/v) glucose served as a control non-CR culture for defining the metabolic patterns created by the CR and *tor1Δ* geroprotectors. A WT strain culture that initially contained 0.2% (w/v) glucose without LCA served as a control CR culture for defining the metabolic pattern created by the LCA geroprotector. Normalized data for three adenosine phosphate nucleotides (i.e., ATP, ADP and AMP) identified in age-matched cells were used to create the principal component analysis (PCA) plots comparing the patterns created by the three different geroprotectors. Yeast recovered on days 17 and 21 were not examined by this PCA because ATP was not detected in WT cells cultured under CR or non-CR conditions without LCA. Data of 2 independent experiments, each being performed twice, are presented.

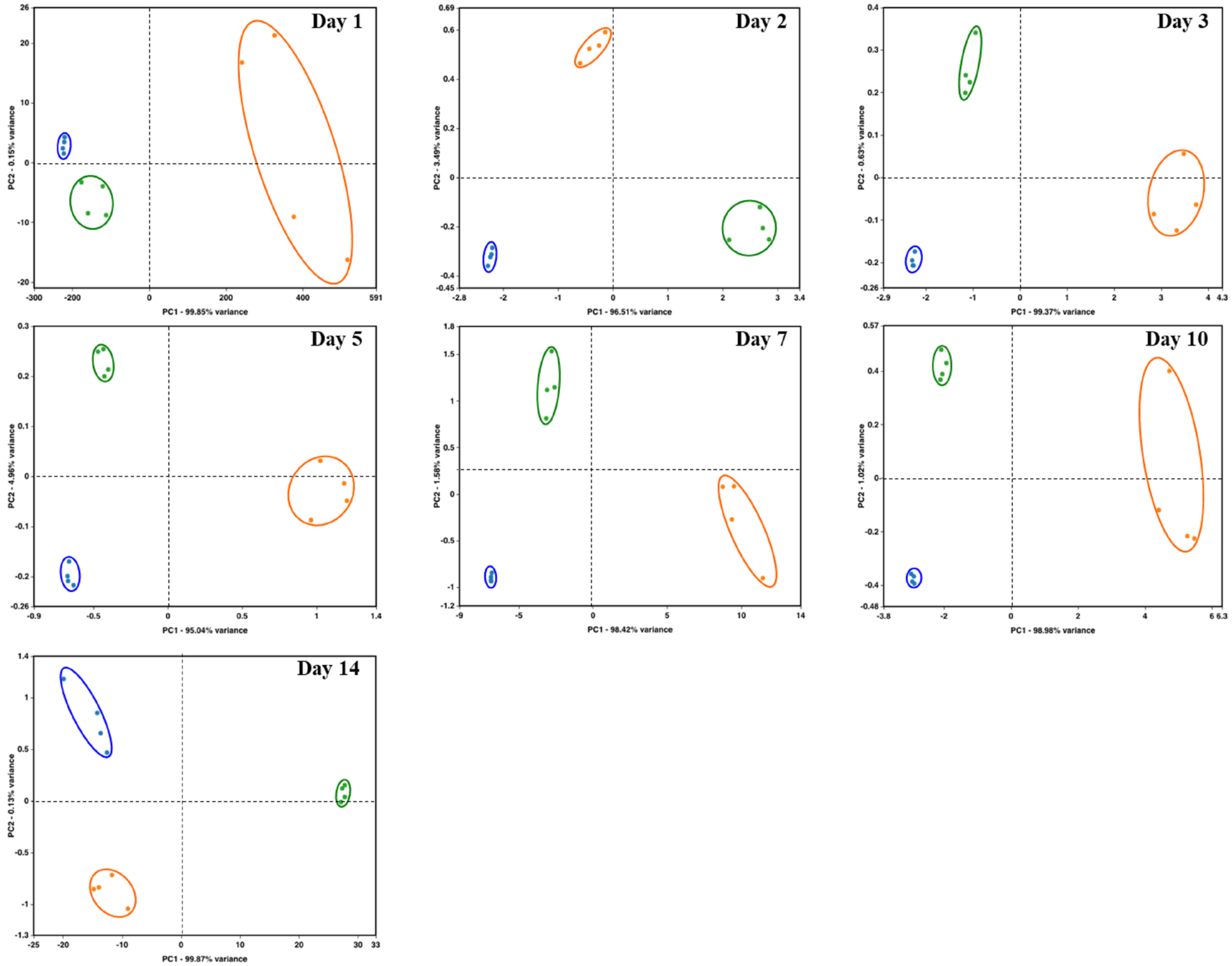

● CR-specific pattern of the ADP:ATP and AMP:ATP ratios ● *tor1Δ*-specific pattern of the ADP:ATP and AMP:ATP ratios ● LCA-specific pattern of the ADP:ATP and AMP:ATP ratios

Supplementary Figure 5: CR, *tor1Δ* and LCA differently affect the ADP:ATP and AMP:ATP ratios at diverse phases of yeast chronological aging. The WT strain BY4742 was cultured in the nutrient-rich YP medium initially containing 2% (w/v) glucose (non-CR conditions), 0.2% (w/v) glucose (CR conditions) or 0.2% (w/v) glucose and 50 μM LCA (CR + LCA conditions). The *tor1Δ* mutant strain in the BY4742 genetic background was cultured in nutrient-rich YP medium initially containing 2% (w/v) glucose (non-CR conditions). Cell aliquots for metabolic activity quenching and metabolite extraction were collected on days 1, 2, 3, 5, 7, 10, 14, 17 and 21 of culturing. The use of LC-MS/MS to identify and quantitate the intracellular water-soluble metabolites is described in Materials and Methods. A WT strain culture that initially contained 2% (w/v) glucose served as a control non-CR culture for defining the metabolic patterns created by the CR and *tor1Δ* geroprotectors. A WT strain culture that initially contained 0.2% (w/v) glucose without LCA served as a control CR culture for defining the metabolic pattern created by the LCA geroprotector. Normalized data for the ADP:ATP and AMP:ATP ratios identified in age-matched cells were used to create the principal component analysis (PCA) plots comparing the patterns created by the three different geroprotectors. Yeast recovered on days 17 and 21 were not examined by this PCA because ATP was not detected in WT cells cultured under CR or non-CR conditions without LCA. Data of 2 independent experiments, each being performed twice, are presented.

Supplementary Table 1: The liquid chromatography gradient program used for the chromatographic separation of extracted metabolites

Time (min)	Flow rate (ml/min)	Relative concentrations (%) of solvents A and B	
		A	B
0.5	0.25	5	95
26	0.25	40	60
30	0.25	70	30
31	0.25	70	30
31.1	0.4	5	95
43.9	0.4	5	95
44	0.25	5	95
45	0.25	5	95

Solvent A: a 95:5 (v/v) mixture of nanopure water with acetonitrile (respectively) containing 20 mM ammonium acetate. Solvent B: acetonitrile. Nanopure water and acetonitrile were of the liquid chromatography-mass spectrometry grade.

Supplementary Table 2: The mass spectrometer settings for analyzing primary ions of the metabolites separated by LC

**Full scan mass range (dalton)**	70–900
**FTMS full scan resolution**	6.0×10^4
**FTMS HCD resolution**	7500
**FTMS full scan AGC target**	1.0×10^6
**FTMS MSn AGC target**	5.0×10^4
**Ion trap (LTQ) MSn AGC target**	1.0×10^4
**Ion source type**	HESI
**Capillary temperature (°C)**	275
**Source heater temperature (°C)**	250
**Sheath gas flow**	10
**Aux gas flow**	5

Abbreviations: AGC, automatic gain control; FTMS, Fourier transform mass spectrometry; HCD, higher-energy collisional dissociation; HESI, heated electrospray ionization; LTQ, linear trap quadrupole; MSn, multi-stage mass spectrometry.

Supplementary Table 3: The mass spectrometer settings for detecting secondary ions of the metabolites separated by LC

**Instrument polarity**	positive/negative
**Activation type**	CID/HCD
**Minimum signal required**	5000
**Isolation width**	2
**Normalized collision energies for CID**	35, 60
**Normalized collision energies for HCD**	35, 45, 55
**Default charge state**	1
**Activation time for CID (ms)**	10, 30
**Activation time for HCD (ms)**	10
**Number of MS/MS events in CID**	top 3, top 5, top 10
**Number of MS/MS events in HCD**	top 5
**Number of micro scans used in both HCD and CID**	1

Abbreviations: CID, collision-induced dissociation; HCD, higher-energy collisional dissociation; ms, milliseconds; MS/MS, tandem mass spectrometry.

Supplementary Table 4: A list of the water-soluble metabolites identified and quantitated in this study. See Supplementary Table 4
