# Supplementary material for: Caloric restriction creates a metabolic pattern of chronological aging delay that in budding yeast differs from the metabolic design established by two other geroprotectors
Source: Oncotarget. 2021 Mar 30;12(7):608–25. doi: 10.18632/oncotarget.27926 (PMC8021023; doi:10.18632/oncotarget.27926)
Supplement: Supplementary file 2 [file oncotarget-12-608-s002.docx]

**Supplementary Table 4: A list of the water-soluble metabolites identified and quantitated in this study**

| **Metabolite’s name** |
| --- |
| Lauric acid |
| Nicotinamide |
| Cytosine |
| Tridecylic acid |
| Adenine |
| N6-Acetyl-lysine |
| Nicotinamide adenine dinucleotide (NAD+) |
| Aspartic acid |
| N-Acetyl-glutamic acid |
| 4-Guanidinobutyric acid |
| Cytidine 5'-diphosphocholine |
| Ethionine |
| 5-Aminovaleric_acid |
| Inosine |
| Riboflavin |
| gamma-Glu-Glu |
| Ureidoisobutyric acid |
| Guanosine |
| Glutamic acid |
| 2-Hydroxyphenylalanine |
| (Hydroxymethyl)phosphonic acid |
| Xanthosine |
| Oleamide |
| Acetyl-carnitine |
| Caprylic acid |
| Maleic acid |
| D-Glucosamine 6-phosphate |
| gamma-Glu-Gln |
| 5-Hydroxy-tryptophan |
| S-Adenosyl-homocysteine |
| Nicotinamide adenine dinucleotide_(NADH) |
| Homoisocitric acid |
| Tryptophan |
| 8-Hydroxyquinoline |
| Cysteine |
| Dopamine 3-O-sulfate |
| Ornithine |
| Prolinamide |
| Cysteinylglycine |
| Aceanthrenequinone |
| Pidotimod |
| Mannitol |
| 5-Aminolevulinate |
| Aminocarb |
| Leu-Leu |
| Cystathionine |
| Phosphonoacetate |
| Uridine-5'-triphosphate_(UTP) |
| 12-Hydroxydodecanoic_acid |
| Malic acid |
| Argininosuccinic acid |
| 2-Furoic_acid |
| 5-Nitrilonorvaline |
| Glutamine |
| Pyroglutamic acid |
| 3-Hydroxy-proline |
| Nicotinic acid mononucleotide |
| Creatine |
| D-Erythrose_4-phosphate |
| Inosine 5'-monophosphate_(IMP) |
| Fosthiazate |
| Deoxyguanosine diphosphate (dGDP) |
| Succinic anhydride |
| Uridine 5'-diphosphate (UDP) |
| 5-Aminopentanamide |
| Carnosine |
| Flavin mononucleotide (FMN) |
| N6, N6, N6-Trimethyl-lysine |
| Dodecanedioic acid |
| b-Ala-Lys |
| 2-Aminobutyric acid |
| Erythritol |
| alpha-Glycerylphosphorylcholine |
| Lysine |
| Pipecolic acid |
| N-Methylhexanamide |
| Deoxycytidine monophosphate (dCMP) |
| Pyridostigmine |
| Leucineamide |
| Acetyl Proline |
| 1-Methylimidazoleacetic acid |
| 4-Methylene-glutamine |
| Adenosine triphosphate (ATP) |
| Orotidine 5'-monophosphate |
| Lysopine |
| Glycine |
| S-Adenosylmethionine |
| Citrulline |
| 3-Phosphoglyceric acid |
| Nicotinic acid |
| N-Acetyl-histidine |
| N6-Methyl-lysine |
| N, N-Dimethylarginine |
| Phenylalanine |
| Benzaldehyde |
| Betaine |
| Acetylarginine |
| Glycerol 3-phosphate |
| 2-Aminoadipic acid |
| D-glucose-6-phosphate |
| Arginine |
| Amobarbital |
| Creatinine |
| Threonic acid |
| 1-phenethylamine |
| Butobarbital |
| Saccharopine |
| Epiguanine |
| Proline |
| N-(4-Oxobutyl)-glutamine |
| Methionine |
| alpha-Kainic acid |
| Cytidine |
| 7-Methylguanine |
| Choline O-Sulfate |
| Asparagine |
| Glyceraldehyde 3-phosphate |
| Phosphoenolpyruvic acid |
| Xanthine |
| Biocytin |
| β-Nicotinamide mononucleotide |
| Itaconic acid |
| Cyclic ADP-ribose |
| N5-Methylglutamine |
| Trimethadione |
| 2'-Deoxyadenosine |
| Guanosine triphosphate (GTP) |
| Sphingosine (d18:1) |
| Adenosine 5'-monophosphate (AMP) |
| Guanine |
| Glutathione oxidized |
| Myristic acid |
| Oxazolidinone |
| 4-Hydroxybenzaldehyde |
| 4-Coumaric acid |
| Imidazolelactic acid |
| n-Heptanoic acid |
| Pentadecanoic acid |
| Stearic acid |
| Afegostat |
| Ethyl myristate |
| D-Xylonic acid |
| Homoserine |
| Alanine |
| Valine |
| Cycloheximide |
| Triphenylphosphine oxide |
| 3-Buten-1-amine |
| Isoleucine |
| Guanosine monophosphate (GMP) |
| Putaminoxin |
| Palmitoleic acid |
| Guanosine 5'-diphosphate (GDP) |
| Fosfosal |
| NADPH |
| 4-Vinylcyclohexene |
| Nonanoic acid |
| Erucamide |
| Flavin adenine dinucleotide (FAD+) |
| N-Acetylcysteine |
| Thiamine |
| Histidine |
| Decanoic acid |
| Penicillamine |
| Ceramide |
| Ethenzamide |
| Serine |
| Naphthylamine |
| Tiglic acid |
| Uridine |
| 4-Aminonicotinic_acid |
| Tyrosine |
| 2'-Deoxyguanosine 5'-monophosphate (dGMP) |
| NADP+ |
| Cytidine 5'-monophosphate |
| Pentabamate |
| Cytidine diphosphate (CDP) |
| 9-Oxononanoic acid |
| Uridine monophosphate (UMP) |
| Lignoceric acid |
| Lactic acid |
| Dodecatrienol |
| Hypoxanthine |
| Dihydroorotic acid |
| Adenosine diphosphate (ADP) |
| Cytidine 5'-triphosphate |
| Myristyl sulfate |
| 3-Hydroxydecanoic acid |
| Acetylcholine |
| Carnitine |
| Leucine |
| Alanyl-proline |
| Lentiginosine |
